# CHIASMA: Advancing chemicals and materials safety and sustainability assessments through innovative integration of in vitro and in silico (new approach) methodologies

**DOI:** 10.1016/j.csbj.2025.11.032

**Published:** 2025-11-16

**Authors:** Pamina Weber, Emma Arnesdotter, Nour Attar, Steffi Friedrichs, Christian Seitz, Riju Roy Chowdhury, Katharina Koch, Tina Buerki-Thurnherr, Beatrice Brugger, Peter Wick, Jack Morikka, Angela Serra, Lorenzo Capini, Dario Greco, Valentina Lacconi, Adriana Scattareggia Marchese, Luisa Campagnolo, Thomas E. Exner, Maja Brajnik, Émilie Brun, Iseult Lynch, Dimitris Zouraris, Dimitris Mintis, Andreas Tsoumanis, Antreas Afantitis, Georgia Melagraki, Vasileios Minadakis, Paraskevi Papakyriakopoulou, Periklis Tsiros, Elisa Thépaut, Haralambos Sarimveis, Marc Majó, Roland Hischier, Ishita Virmani, Martin Paparella, Simona Kavaliauskiene, Amin Sayyari, Romain Fontaine, Mette Helen Bjørge Müller, Eunseo Lee, Zayakhuu Gerelkhuu, Tae Hyun Yoon, Duygu Turan-Sorhun, Winfried Neuhaus, Tommaso Serchi

**Affiliations:** a Luxembourg Institute of Science and Technology Grand Duchy of Luxembourg, Luxembourg; b AcumenIST SRL, Rue Fétis 19, Etterbeek, Brussels 10401, Belgium; c IUF - Leibniz Research Institute for Environmental Medicine, Düsseldorf, Germany; d DNTOX GmbH, Düsseldorf, Germany; e Swiss Federal Laboratories for Materials Science and Technology (Empa), Lerchenfeldstrasse 5, St. Gallen 9014, Switzerland; f Faculty of Medicine and Health Technology, Tampere University, Tampere, Finland; g Tampere Institute for Advanced Study, Tampere University, Tampere, Finland; h Division of Pharmaceutical Biosciences, Faculty of Pharmacy, University of Helsinki, Helsinki, Finland; i Dept. Biomedicine and Prevention, University of Rome “Tor Vergata”, Rome, Italy; j Seven Past Nine d.o.o., Hribljane 10, Cerknica 1380, Slovenia; k School of Geography, Earth and Environmental Sciences, University of Birmingham, Edgbaston, Birmingham B15 2TT, United Kingdom; l NovaMechanics Ltd, Nicosia, Cyprus; m Division of Physical Sciences and Applications, Hellenic Military Academy, Vari 16672, Greece; n School of Chemical Engineering, National Technical University of Athens, Athens, Greece; o Institute of Medical Biochemistry, Medical University Innsbruck, Innsbruck, Austria; p Department of Production Animal Clinical Sciences, Faculty of Veterinary Medicine, Norwegian University of Life Sciences, Ås, Norway; q Department of Chemistry, Hanyang University, Seoul 04763, Republic of Korea; r Institute of Next Generation Material Design, Hanyang University, Seoul 04763, South Korea; s Competence Unit Molecular Diagnostics, AIT Austrian Institute of Technology GmbH, Vienna, Austria; t Faculty of Medicine and Dentistry, Danube Private University (DPU), Krems, Austria; u Department of Pharmacy, Frederick Universiy, Nicosia 1036, Cyprus

**Keywords:** New Approach Methodologies (NAMs), Next generation Safety Assessment (NGSA), 3Rs (Replacement, Reduction, Refinement), chemical safety assessment, Safe and Sustainable by Design (SSbD), PFAS, (nano-)pesticides, advanced materials, European Green Deal

## Abstract

Traditional *in vivo* methodologies have long formed the foundation of chemical and material safety assessment, yet they are increasingly inadequate to meet modern regulatory, ethical, and sustainability demands. These conventional approaches are resource-intensive, ethically questionable, and often fail to accurately predict human or environmental toxicity, particularly for emerging pollutants such as PFAS, (nano-) pesticides, and 2D materials. In response, the EU has launched initiatives like the Chemical Strategy for Sustainability and the Zero Pollution Action Plan under the European Green Deal to promote innovation in safer, and more sustainable chemicals. Central to this transformation is the Safe and Sustainable by Design (SSbD) framework, developed by the European Commission’s Joint Research Center, which provides structured methodologies and metrics to integrate safety and sustainability into material innovation from the earliest stages of design. Building on this vision, the CHIASMA project aims to advance Next generation Safety Assessment (NGSA) by developing innovative New Approach Methodologies (NAMs) that combine experimental, computational, and Life Cycle Assessment (LCA) tools. Focusing on key biological systems and exposure routes, CHIASMA integrates Artificial Intelligence (AI), Machine Learning (ML), and Knowledge Graph (KG) technologies to enhance data interoperability and predictive accuracy. By embedding FAIR data principles and aligning with Good Laboratory Practice (GLP) standards, CHIASMA promotes transparency and regulatory acceptance. Fully aligned with SSbD principles, CHIASMA establishes a digital, interoperable infrastructure for predictive safety evaluation, that leverage on state-of-art experimental New Approach Methodologies (NAMs) bridging critical data gaps and supporting the transition towards sustainable, science-driven, and ethically responsible chemical and material innovation in Europe and beyond.

## Graphical Abstract

**Figure:**
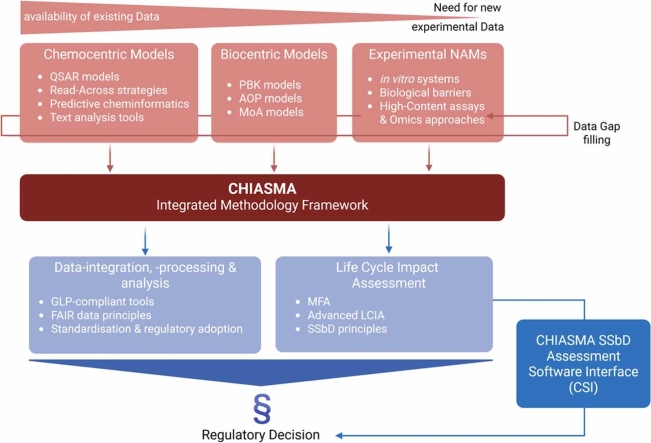
Illustration of the CHIASMA R&I approach to testing and assessment of materials using an iterative approach based on the integration of chemocentric, biocentric and new experimental models into a conceptual framework of data-integration and -processing.

## 1 Introduction

The safety assessment of chemicals and materials has traditionally relied on *in vivo* animal testing, forming the backbone of existing regulatory frameworks. Although these approaches have provided valuable knowledge, they are increasingly recognized as insufficient for addressing existing regulatory and sustainability challenges [1]. Their limitations are multifaceted [2]. They are resource-intensive, requiring substantial investment of time, funding, and logistics, which often delays safety evaluations [3]. Ethical concerns surrounding animal testing have also intensified, driven by societal expectations and regulatory commitments to the 3Rs (Replacement, Reduction, Refinement) [4]. Moreover, inter-species differences frequently compromise the relevance of animal models for predicting human toxicity. Traditional approaches also fail to align with modern sustainability objectives, as they rarely integrate human health protection with environmental sustainability.

These weaknesses are particularly evident when assessing emerging or advanced materials, such as per- and polyfluoroalkyl substances (PFAS),^1^ nano-pesticides, and two-dimensional (2D) materials. Advanced materials, engineered for enhanced strength, durability, responsiveness, or functionality compared to conventional materials, exhibit complex and dynamic properties that challenge conventional *in vivo* assessment methods. Consequently, critical data gaps remain regarding bioaccumulation, chronic toxicity, and long-term environmental impact [5].

In response to societal demands and technical needs, the European Union (EU) has launched several initiatives, including the Chemical Strategy for Sustainability (CSS) [6] and the Zero Pollution Action Plan [7], both integral to the European Green Deal [8]. These initiatives aim to achieve climate neutrality and a resource-efficient, non-toxic environment by reducing pollution across air, water, and soil, while fostering innovation in the design, management and testing of safer and more sustainable chemicals and materials. They also recognize the growing importance of Artificial Intelligence (AI) and digital technologies in advancing data-driven chemicals risk assessment.

To guide this transformation, the European Commission’s Joint Research Centre (JRC) developed the SSbD framework [9], which provides a high-level methodology to embed safety and sustainability considerations from the earliest stages of innovation. The SSbD approach promotes a paradigm shift in how substances are developed, produced, evaluated, and managed, encouraging early integration of hazard reduction, environmental performance, circularity, and functionality [10]. While the SSbD principles articulate a vision for inherently safe and sustainable materials and products, the framework translates this vision into practice through measurable criteria, indicators, and methodologies for safety and sustainability assessment, including risk assessment, life-cycle analysis, and impact evaluation tools aligned with EU regulations.

To guide this transformation, the European Commission’s JRC developed the SSbD framework [9], which provides a high-level methodology to embed safety and sustainability considerations from the earliest stages of innovation. The SSbD approach promotes a paradigm shift in how substances are developed, produced, evaluated, and managed, encouraging early integration of hazard reduction, environmental performance, circularity, and functionality [10]. While the SSbD principles articulate a vision for inherently safe and sustainable materials and products, the framework translates this vision into practice through measurable criteria, indicators, and methodologies for safety and sustainability assessment, including risk assessment, life-cycle analysis, and impact evaluation tools aligned with EU regulations.

The SSbD framework also supports the objectives of the EU Industrial Strategy and the Circular Economy Action Plan (CEAP) [11], ensuring that material innovation contributes to sustainable production and consumption across Europe. By embedding safety and sustainability into research and industrial development, SSbD not only mitigates risks to human health and the environment but also accelerates the deployment of greener, more competitive technologies.

Despite these efforts, the field continues to face critical challenges. Current tools often lack the mechanistic insights needed to predict long-term biological and ecological outcomes, such as chronic toxicity and ecosystem disruptions. Regulatory assessment is further hindered by limited information on Adverse Outcome Pathways (AOPs), which are essential for connecting molecular mechanisms to observable adverse effects [12]. In addition, the insufficient integration of *in silico* and *in vitro* methods has led to fragmented data landscapes, undermining the coherence and reliability of predictive models and sowing their acceptance in regulatory contexts [13].

The project CHIASMA addresses these challenges by developing innovative methodologies and frameworks for chemical and material safety assessment. The work presented here is guided by the following working hypothesis, which frame the purpose and scope of CHIASMA:

(i) A tiered integration of chemocentric models, biocentric models and optimized experimental NAMs can provide decision-relevant evidence for key REACH- and CLP-relevant endpoints for representative groups of substances (PFAS, (nano-)pesticides, and 2D materials), while substantially reducing reliance on traditional animal testing.
(ii) A harmonised digital backbone, combining knowledge graphs, PBK models, OMICS-based mechanistic information, and AI/ML approaches within a common SSbD Assessment platform, will increase the coherence, transparency, and mechanistic interpretability of Next Generation Safety Assessment compared with the current, more fragmented use of individual NAMs.
(iii) Feeding CHIASMA-derived human- and eco-toxicity information (including refined effect factors and characterization factors) into MFA- and LCA-based Life Cycle Impact Assessment will lead to different prioritisation and ranking of materials and use scenarios than assessments relying on conventional, animal-based toxicity data alone, thereby supporting Safe-and-Sustainable-by-Design decisions.

From the one side, CHIASMA aims at developing an integrated framework that leverages and a multi-tiered approach combining *in silico* and experimental tools to enable NGRA in regulatory toxicology. By developing and integrating experimental and computational high-quality NAMs [14], [15], [16], CHIASMA aims to overcome the limitations of traditional methods. From the side of experimental NAMs, CHIASMA focuses on key biological systems (liver, brain, endocrine, skin, intestine, lung, placenta, and kidneys), while addressing key routes of exposure such as inhalation, ingestion, and dermal absorption. By integrating these elements CHIASMA establishes a robust foundation for predictive safety evaluation. This approach, despite not addressing the totality of the field, will show the strengths and drawbacks of the approach, paving the way for further advancements in NGRA and in SSbD in general.

At the core of the project’s vision lies the seamless integration of advanced digital and analytical technologies into a cohesive, interoperable framework that supports innovation, informed decision-making, and sustainable assessment across disciplines. Central to this approach is the use of knowledge graphs, which structure and interlink complex, heterogeneous datasets in a machine-readable format, thereby enhancing data interoperability and accessibility. Machine Learning (ML) and AI provide predictive and adaptive capabilities, enabling the system to identify patterns, optimize analytical performance, and support evidence-based decisions throughout the product and process lifecycle. Complementing these digital tools, the Life Cycle Assessment (LCA) methodology offers a quantitative foundation for evaluating environmental impacts, ensuring that sustainability considerations are embedded from the earliest stages of design and development.

By integrating these components, CHIASMA establishes a robust digital infrastructure that strengthens the predictive power of safety assessments and bridges critical knowledge gaps related to emerging pollutants and advanced materials, such as nanomaterials (e.g., titanium dioxide, graphene, carbon nanotubes), smart polymers, and engineered biomaterials. Any remaining data gaps are addressed through the deployment of *in vitro* NAMs developed within the project, ensuring comprehensive coverage of both human and environmental safety aspects.

CHIASMA’s methodological design is driven by the overarching goal of advancing societal sustainability objectives. Transparency and data reusability are ensured through the implementation of FAIR principles (Findable, Accessible, Interoperable, Reusable) [17], while technical rigour and reliability are maintained through alignment with Good Laboratory Practice (GLP) standards. This integrated strategy promotes transparency, scalability, and facilitates broader regulatory acceptance at both European and global levels.

In this context, CHIASMA represents a pivotal step towards transforming safety and sustainability assessment, addressing the evolving needs of regulators, industry, and society, and accelerating the transition towards Next Generation Safety Assessment (NGSA).

## 2 Project Description

The CHIASMA project aims to revolutionize chemical and material safety assessment by developing a multi-tiered, animal-free framework for regulatory applications. The CHIASMA Framework leverages in vitro and in silico New Approach Methodologies (NAMs) to support the development of improved Life Cycle Impact Assessment (LCIA) approaches and strategies, enabling Next Generation Risk Assessment (NGRA). Ultimately, these elements will be integrated into a comprehensive, user-friendly, and interoperable framework aligned with the EU’s SSbD principles and regulatory frameworks. The CHIASMA framework is designed to complement key European legislation, including the Registration, Evaluation, Authorisation and Restriction of Chemicals (REACH) and the Classification, Labelling and Packaging (CLP) regulations, as well as other relevant policy instruments.

The resulting CHIASMA SSbD Framework, covering both safety and environmental dimensions, is built on the coherent development, refinement, and demonstration of NAMs derived from both in silico and experimental methods. This integrative approach aims to empower risk assessors, SMEs, large enterprises, and regulators to address REACH- and CLP-relevant endpoints through human-relevant, 3R-compliant methodologies. The NAMs developed, refined, and demonstrated within CHIASMA are conceived as a versatile “Swiss army knife” of regulatory science, capable of generating new data, reprocessing existing datasets, and combining in silico, in vitro, and in chemico approaches. These methods can be applied individually or as part of Integrated Approaches to Testing and Assessment (IATAs) to produce regulatory-relevant outcomes within the EU SSbD framework.

As formal validation cannot be achieved within the project timeframe, the CHIASMA SSbD Framework and its underlying *in silico* and experimental NAMs will undergo “in-project” validation using three groups of socially and environmentally relevant substances:

a) Demo-Case 1:Polyfluoroalkyl Substances (PFAS)
b) Demo-Case 2: (nano)-pesticides
c) Demo-Case 3: 2D materials for energy applications.

These groups were selected due to their high potential for human health and environmental impacts, persistence, and extensive industrial use, which have generated considerable societal concern.

CHIASMA builds upon many years of European and international Research and Innovation (R&I) in chemical and nano-safety, computational model development, and advanced biological model design for predicting safety and environmental effects. It also integrates conceptual frameworks established through collaborative initiatives in regulatory toxicology and sustainability science.

**Fig. 1:**
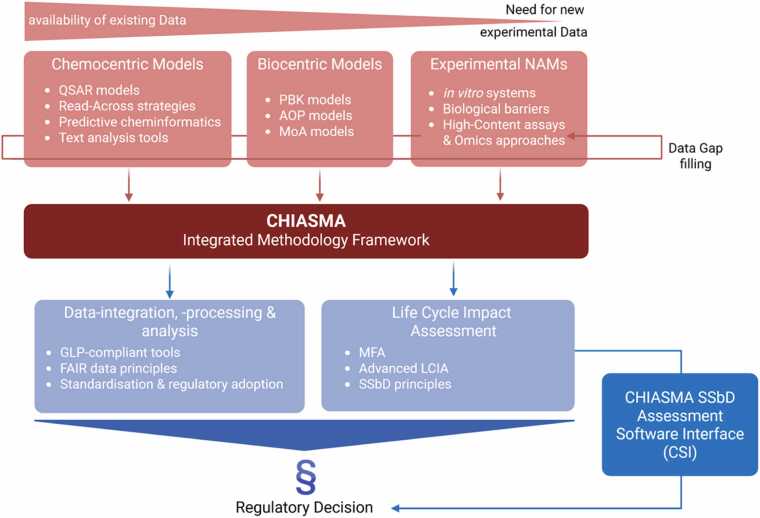
Overview of the CHIASMA R&I approach to testing and assessment of materials using an iterative approach based on the integration of chemocentric, biocentric and new experimental models into a conceptual framework of data-integration and -processing. The figure was created with BioRender^Ⓡ^ (biorender.com/).

The core concept of CHIASMA, illustrated in Fig. 1, centres on the establishment of an integrated methodology for chemical safety and sustainability assessment. This methodology maximizes the use of existing data, refines hazard prediction, and minimizes reliance on traditional animal testing. The CHIASMA SSbD Assessment provides regulators with a structured, iterative, and multi-step process grounded in NGSA principles. It is built upon three complementary pillars: chemocentric models**,** biocentric models, and experimental NAMs (including in vitro systems and OMICS-based approaches).

The CHIASMA framework is structured around three complementary pillars: chemocentric models**,** biocentric models**,** and optimized experimental NAMs. Together, these components form an integrated, interoperable system for chemical and material safety assessment.

1. Chemocentric models, powered by state-of-art artificial intelligence, include:

• Predictive models describing the behaviour and properties of chemical systems.
• Quantitative Structure-Activity Relationship (QSAR) models linking chemical features to biological effects.
• Material Flow Analyses (MFA) for estimating chemical and material releases across product life cycles.
• Text-mining tools for processing and extracting relevant information from large volumes of unstructured text.
• Knowledge Graph (KG) for structuring and interlinking existing data, enabling inference of new relationships and conclusions.
• Read-Across approaches to predict properties of target substances based on data from structurally or functionally analogous compounds.

2. Biocentric models, consisting of:

• Physiologically Based Kinetic (PBK) models to simulate the absorption, distribution, metabolism, and excretion (ADME) of chemicals in biological systems.
• AOP models providing a systematic understanding of the Key Events (KEs) and Molecular Initiating Events (MIEs) leading to adverse outcomes.
• Mechanism of Action (MoA) models describing molecular and cellular interactions upon exposure, helping to identify affected targets, pathways, and processes.

3. Optimised experimental NAMs, representing relevant human biological systems, include:

• Human organs and systems such as the brain, reproductive/developmental and endocrine systems, kidney, and liver.
• Internal biological barriers, including Blood-Brain-Barrier (BBB) and Blood-Placenta-Barrier (BPB).
• External barrier, such as the lung alveolar interface, skin, and small intestine.

All the gathered data and results are then subjected to data integration, processing and analysis, as well as integration into LCIA modules. Within the first of these two modules, we make data, results, methods and SOPs as open, transparent and transferable as possible (“*FAIRification*” and “*GLP-ification*” activities). During the CHIASMA’s lifetime, the *in silico* and *in vitro* NAMs will be further advanced and tested, with the aim of initiating the formal validation process within the project’s lifespan or shortly after its conclusion. Formal validation ensure that MAD (Mutual Acceptance of Data) principles are applicable to the developed methods, further boosting the penetration of the developed NAMs, their regulatory uptake, and commercial exploitability. Formal regulatory validation is initiated by submission of Standard Project Submission Form (SPSF) to the OECD (Organization for Economic Co-Operation and Development), Working Party for the Test Guideline Program (WNT) committee, or the (pre)validation forms to EURL-ECVAM (EU Reference Laboratory for Alternatives to Animal Testing).

In parallel, the LCIA module will extend existing toxicity assessment approaches, such as the USEtox method, to achieve a more comprehensive integration of human and environmental toxicity within LCA. This will ensure that CHIASMA’s digital and experimental tools not only enhance mechanistic understanding but also contribute to sustainability metrics and regulatory decision-making.

The CHIASMA Tiered Approach integrates these concepts into a structured, iterative process aligned with NGSA principles for chemical and material safety and environmental evaluation. At its core lies the extensive reuse and integration of existing human and environmental toxicity data, which form the foundation for the initial chemocentric analyses. This stage employs automated data retrieval through text-mining and KG technologies, followed by in silico methods such as QSAR modelling**,** read-across techniques, and structural alerts to infer potential hazards and relevant chemical–biological interactions.

However, the predictive power of chemocentric models remains constrained by data availability and quality, as well as their limited ability to capture biological variability, including metabolism, reactivity, and bioavailability, which may result in uncertainty or incomplete safety conclusions. When chemocentric results are insufficient or inconclusive, the CHIASMA framework escalates the assessment by integrating biocentric approaches. These include PBK models to simulate ADME processes, and AOP/MoA frameworks that employ computational biology, systems modelling, and machine learning to explore complex mechanistic responses underlying adverse outcomes. The combined outputs from these models are critically evaluated against regulatory criteria to assess consistency, robustness, and biological plausibility.

If uncertainty persists, owing to limited pathway validation, insufficient training data, or challenges in biological extrapolation, the assessment advances to the experimental NAM tier. This stage deploys innovative non-animal methodologies, including 3D cell cultures, organ-on-chip systems, high-throughput screening assays, and ex vivo models. These systems are strategically selected to represent key human exposure routes (inhalation, ingestion, and dermal absorption) and target organs or barriers (liver, kidney, skin, intestine, lung, brain, and placenta). By characterizing compound-specific kinetics across biological interfaces, these in vitro systems refine PBK models and reinforce computational predictions, thereby improving the reliability of integrated safety evaluations.

While CHIASMA is committed to advancing animal-free testing strategies, it acknowledges that certain complex endpoints, such as specific aspects of developmental and reproductive toxicity, may still require complementary testing. In such cases, the use of alternative *in vivo* systems based on less sentient species (e.g., fish embryo models) is proposed, in alignment with current ethical and scientific standards and consistent with the principles of the 3Rs (Replacement, Reduction, Refinement).

CHIASMA’s NAMs are developed across two main categories:

• Computational NAMs: *in silico* tools including QSAR and PBK models, read-across tools, AOP networks, and knowledge graph-based inference systems.
• Experimental NAMs: advanced *in vitro* and *ex vivo* systems.

These tools are designed to generate regulatory-relevant data, with validation pathways informed by literature reviews and aligned with scientific and regulatory standards.

Despite extensive research, many toxicological uncertainties remain, particularly regarding long-term exposure and environmental persistence. To address these uncertainties, it is essential to adopt assessment approaches that account for both exposure and toxicity throughout the entire material life cycle. Within CHIASMA, established methodologies such as MFA and LCA will be applied: MFA enables the quantification of material stocks at large territorial scales, while LCA assesses impacts at the product level.

For this, LCA evaluates the potential impacts pf the product or process using the inventory data and the so-called Characterization Factors (CFs), that allow to translate inventory flows in to impacts [18]. There are various LCIA methods that differ in their selection of impact categories and characterization models, as well as their final aggregation level [19], [20]. To encourage the use of standardized methods for measuring and communicating the environmental performance of products, the EC recommends the Product Environmental Footprint (PEF) method [21]. With its activities, CHIASMA improves the toxicity-related assessments (through the expansion and refinement of the USEtox model), enabling a more comprehensive and complete sustainability assessment.

To address limitations of *in vitro* systems for long-term health outcomes, CHIASMA is developing endpoint-specific, predictive biomarkers for MIE and KE, such as genes with significantly regulated expression and epigenetic markers for conditions like lung fibrosis, Parkinsonian motor deficits, and reproductive toxicity. The biomarkers are first derived using well-known, data-rich toxicants (e.g. acrolein, bleomycin, methyl methane sulfonate (MMS)) that allow hypothesis driven studies as well as holistic studies, before being applied to data-poor substances.

CHIASMA’s methodologies are demonstrated through real-world case studies on PFAS, (nano-) pesticides, and 2D materials for energy applications, each chosen for their environmental risk relevance and regulatory challenges [22], [23], [24], [25], [26], [27], [28].

### 2.1 Per- and polyfluoralkyl substances (PFAS)

Perfluorooctanesulfonic acid (PFOS) and perfluorooctanoic acid (PFOA), both belonging to the PFAS chemical group, are widely used by industry and in consumers’ products. PFAS are extremely persistent in the environment and exert various toxic effects by exceeding the tolerable weekly intake for certain populations in the EU [29]. The ubiquitous use of this large and chemically diverse group of synthetic compounds is based on their heatproof, waterproof, and non-stick properties, that make them attractive in many industrial and consumer applications, including non-stick cooking utensils, cleaning products and furniture [30], [31]. They are also widely used in industrial and medical applications such as electroplating, biomedical imaging, firefighting, and textile industries, etc. [32], [33], [34], [35]. However, some PFAS, especially the long-chain compounds such as PFOA and PFOS have been linked to developmental [36], hepatic [37], immune [38], [39], and endocrine toxicity [39], [40], as well as carcinogenic effects [41]. These substances are now banned or strictly regulated, classified as Persistent Organic Pollutants (POPs) under the Stockholm Convention [42], and considered legacy chemicals within the EU regulatory framework. Nonetheless, many other PFAS are still being produced and used. Under REACH and CLP, numerous PFAS are subject to regulatory controls, but due to the structural diversity of PFAS and the continuous development of new variants, a strict regulation remains challenging. While in the past, the most widely used PFAS were the long-chains, the development is now moving more and more towards mid- and short-chain PFAS for which the developed techniques for the removal and destruction are inefficient.

Despite the increasing public and regulatory pressure to eliminate PFAS completely, their unique physicochemical properties make them essential for many applications. Therefore, while being restricted, many PFAS are still authorized for certain applications in cases where no viable alternatives exist.

### 2.2 (Nano-)Pesticides

Used to enhance crop yields, pesticides, including nano-formulations, may pose acute and chronic health risks. Acute exposure to pesticides, mainly via inhalation, ingestion, or dermal contact, causes a significant number of deaths globally each year, kills mainly caused by self-poisoning [43]. Chronic exposure is linked to neurological disorders, cancers, reproductive harm, and immune dysfunction [44], [45], [43]. Many pesticides act as endocrine-disruptive chemicals (EDCs), interfering with hormone synthesis, receptor binding, and thyroid dysfunction. Legacy substances like Dithiothreitol (DTT), Glyphosate, and Vinclozolin mimic or block estrogenic and androgen pathways, contributing to infertility, developmental defects, and altered neurobehavior [46].

Most pesticides persist in soil and water and remain detectable even decades after their use, thereby harming birds, aquatic life, and beneficial microorganisms [47]. Therefore, many substances are restricted or banned under the EU’s REACH and CLP frameworks due to their carcinogenic and reprotoxic properties.

### 2.3 2D material for energy application

2D materials, such as Graphene, Carbon Nanotubes (CNTs), Transition Metal Dichalcogenides (TMDs), and Transition Metal Carbides (MXenes), offer high potential in energy storage and conversion due to their electrical conductivity and mechanical strength. These materials show high surface area, excellent electrical conductivity, and good mechanical properties, making them promising candidates for batteries, supercapacitors, solar cells, and thermoelectric or photovoltaic devices [48]. However, these materials pose potential health and environmental risks. TMDs have been linked to cytotoxicity, oxidative stress *in vitro*, and lung inflammation and fibrosis *in vivo* upon inhalation [49]. Tungsten carbide is known to induce genotoxic effects [50] and has been suggested as positive control for chromosomal damage evaluation by the *in vitro* micronucleus assay [51]. Carbon-based 2D materials, such as Graphene and CNTs, also raise safe concerns. While certain Graphene derivates are generally regarded as well tolerated in specific applications, other forms may induce cytotoxicity and genotoxicity depending on their size, shape, and surface chemistry [52], [53]. CNTs, especially long and rigid nanotubes, are known to induce asbestos-like effects such as lung inflammation and mesothelioma [54] and to interfere with fetal development following maternal exposure [55].

Central to the CHIASMA’s framework is the “CHIASMA SSbD Assessment Common Software Interface (CSI)”, powered by the Enalos Cloud Platform [56], [57] and Jaqpot [58]. The CSI unifies all project components within a single platform, providing consortium members and external users with access to experimental and *in silico* NAMs, data, standard operating procedure (SOPs), quality metrics, and uncertainty analyses. Its user-friendly interface and modular design support data curation, NAM execution, IATA operations, and result visualisation, ensuring seamless interoperability across regulatory and industrial contexts. By integrating legacy tools, adhering to FAIR principles, and implementing GLP-compliant protocols, the CSI facilitates transparency, scalability, and efficient deployment.

**Fig. 2:**
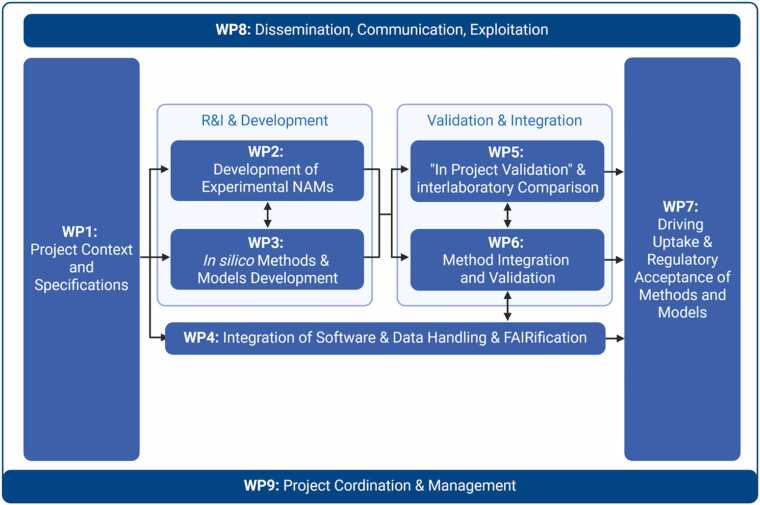
Overview of the CHIASMA Project structure, individual WPs and the workflow and dependencies between them. The figure was created with BioRender^Ⓡ^ (biorender.com/).

The structure of the CHIASMA project is designed to ensure maximum collaboration and alignment of activities to achieve the project’s objectives (Fig. 2).

**Fig. 3:**
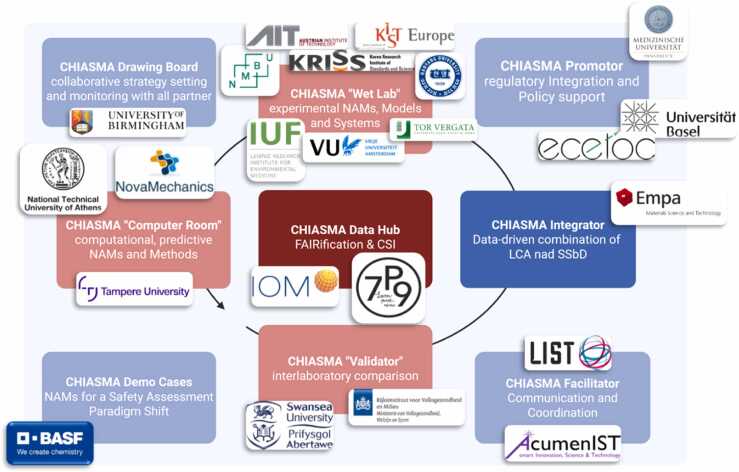
Overview of the workflow and the consortium of the CHIASMA project. The figure was created with BioRenderⓇ (biorender.com/).

To ensure the successful implementation of the CHIASMA project, a consortium of 23 partners from 14 countries was established, bringing together expertise from academia, research institutions, industry, and policy organizations. This diverse composition guarantees a multidisciplinary and cross-sectoral approach to advancing the safety and sustainability assessment of modern chemicals and materials (Fig. 3).

Academic and research institutions, including the Luxembourg Institute of Science and Technology (LIST), Tampere University (TAU), Medical University of Innsbruck (MUI), Leibniz Institute for Environmental Medicine (IUF), Norwegian University of Life Sciences (NMBU), University of Birmingham (UoB), Swansea University (SU), University of Rome Tor Vergata (UniTOV), National Technical University of Athens (NTUA), Austrian Institute of Technology (AIT), Vrije Universiteit Amsterdam (VUA), Swiss Federal Laboratories for Materials Science and Technology (Empa), and Korea Institute of Science and Technology (KIST) Europe, provide cutting-edge expertise in toxicology, environmental science, computational biology, and advanced methodological development and validation.

Within this network, LIST and IUF contribute specialized expertise in human health hazard assessment and exposure science, playing central roles in the refinement and validation of mechanistic test methods. Industry partners, including BASF SE, Seven Past Nine, and NovaMechanics (NovaM), ensure the practical applicability and industrial relevance of CHIASMA’s outputs. Meanwhile, the National Institute for Public Health and the Environment (RIVM), the European Centre for Ecotoxicology and Toxicology of Chemicals (ECETOC), and the University of Basel (UniBAS) provide regulatory and scientific guidance, ensuring alignment with international standards and supporting regulatory acceptance.

The consortium also benefits from a global dimension, with contributions from Hanyang University (HYU) and the Korea Research Institute of Standards and Science (KRISS), which broaden the project’s international collaboration and data harmonization efforts. Finally, Acumenist (AIST) leads the project’s dissemination, communication, and exploitation activities, ensuring the effective translation of scientific outcomes into policy and innovation.

## 3 Impact

### 3.1 Development of Experimental NAMs

The CHIASMA project is dedicated to advancing innovative approaches for the safety and sustainability assessment of chemicals and materials. A major focus lies in the development and optimization of experimental NAMs. CHIASMA aims to advance these methods toward GLP readiness, ensuring their reliability and regulatory applicability. To achieve this, the project evaluates the reproducibility and transferability of each methodology both within and across participating laboratories.

**Fig. 4:**
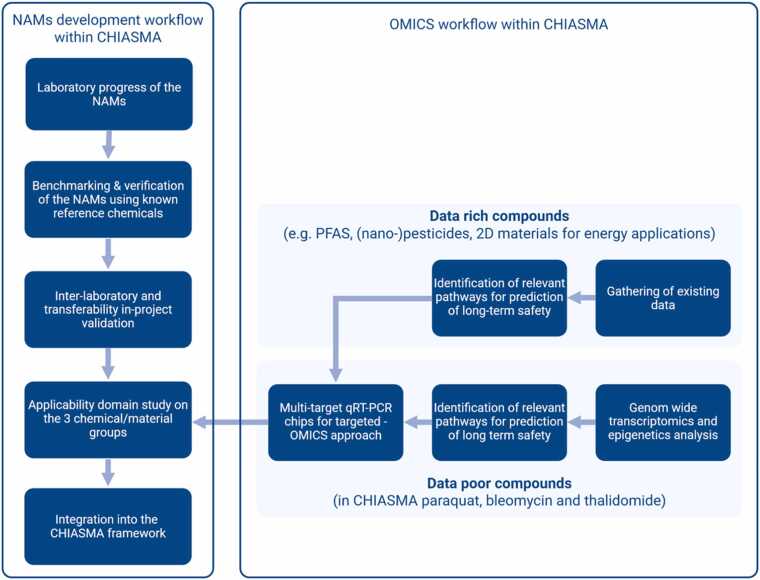
Overview of CHIASMA’s adaptive approach, whose steps depend on the data-richness of the chemical/ material. The figure was created with BioRenderⓇ (biorender.com/).

A central objective of CHIASMA is to establish a robust intra- and inter-consortium validation strategy, designed to support the regulatory acceptance of NAMs and facilitate their eventual inclusion in formal validation processes (Fig. 4). This structured validation framework ensures that the developed methods meet the quality standards required for use in regulatory toxicology and risk assessment.

The experimental NAMs developed within CHIASMA are designed to support the application of the 3Rs principles (Replacement, Reduction, and Refinement) with the overarching goal of minimizing animal testing while maintaining robust and reliable risk assessments. Major regulatory and scientific organizations, including the Organisation for OECD, the European Chemicals Agency (ECHA), the U.S. Food and Drug Administration (FDA), the National Institutes of Health (NIH), the European Medicines Agency (EMA), and the European Food Safety Authority (EFSA), all promote the development and implementation of such methods as scientifically valid alternatives to traditional animal testing. These efforts align with the first two Rs, Replacement and Reduction, by reducing reliance on animal models and enhancing the predictive quality of chemical safety evaluations.

Within CHIASMA, 13 experimental in vitro NAMs are being further developed and demonstrated in alignment with relevant regulatory biological endpoints, as summarized in Table 1. The selection of these models was guided by their capacity to evaluate chemical effects across multiple exposure routes and to provide data suitable for integration with Physiologically Based Pharmacokinetic (PBPK) models.

**Table 1:** List of experimental in vitro NAMs being developed in CHIASMA.

***Experimental in vitro NAMs***		
*Name of the NAM*	*Developer*	*Endpoints*
***Biological Barriers***		
ALIsens® model [59]	LIST	• Cell viability • Acute Toxicity • Respiratory sensitization • Irritation • Genotoxicity (COMET assay) • Genotoxicity (Micronucleus assay)
Skin model [60]	Empa	• Irritation • Sensitization
Intestine model [61]	Empa	• Barrier integrity • Cell viability • Inflammation • Lipid uptake
Placenta model [62]	Empa	• Barrier integrity • Endocrine function • Cell viability • Barrier translocation
Placenta-embryo model [63]	UniTOV	• Placental barrier formation and integrity (indicators: TEER values and markers expression). Barrier translocation of testing substances: • Time-dependent quantification of chemicals in upper and basolateral transwell compartments • Effects on embryo development (indicators: expression of stemness and differentiation markers)
BBB spheroid *in vitro* model [64][65]	AIT	• Barrier integrity • Cell viability • Cell toxicity
BBB Transwell *in vitro* model as alternative [66]	AIT	• Barrier integrity • Cell viability • Cell toxicity • Toxin permeability (depending on availability of analytics)
***Metabolism***		
Liver spheroids [67], [68], [69], [70]	SU	• Chromosomal damage • DNA strand breaks • Point mutations • Gene expression changes • Inflammatory response
Kidney model [71]	VUA	• Barrier integrity • Cell viability • Lactate/Glycolysis OCR/ Mitotox/ Gene changes
***Critical Organs/ processes***		
Neuroendocrine model [72]	NMBU	Nonreproductive toxicity (BPG axis): • Tissue viability (brain and pituitary) • Hormone production • Receptor activation
Gonadal model [73], [74]	NMBU	Reproductive toxicity: • Egg/sperm maturation and viability • Sperm quality • Fertilisation success
Developmental neurotoxicity model (Neurosphere Assay) [75]	IUF	• Human Neural Progenitor Cell (hNPC) proliferation, • Migration of radial glia cells, neurons and oligodendrocytes • Differentiation of neurons and oligodendrocytes • Neurite outgrowth • Mitochondrial activity • Cytotoxicity
Adult neurotoxicity model (hMNR assay, BrainSphere model for Parkison’s disease) [76]	IUF	• Spike-, burst- and network-related parameters based on mixed neuron/glia neural networks • chronic exposure of matured networks (effects on individual neuronal subtypes detectable) • Cytotoxicity
3D Heart organoids	KIST Europe	• Cardiotoxicity • Heart Rate Monitoring
Daphnia magna [77], [78]	KIST Europe	• Cardiotoxicity • Heart Rate Monitoring
HTS Imaging system [79], [80], [81]	KRISS	• Heart Rate Monitoring

The selected experimental systems are grouped into three main categories:

1. Models that represent internal and external barriers:
  Lung
  Skin
  Intestine
  Placenta
  BBB
2. Models that represent organs that are involved in metabolism:
  Liver
  Kidney
3. Models that represent critical organs/processes:

Development

Heart

Brain

Together, these experimental NAMs provide comprehensive coverage of key human physiological systems and exposure routes, supporting the generation of mechanistically relevant, human-centric data for regulatory and safety assessment purposes.

#### 3.1.1 Modular validation approach

CHIASMA adopts a modular validation approach in accordance with the OECD Guidance Document 34 (GD 34) [82], which defines three sequential phases of validation:

1) Test Development
2) Initial pre-validation
3) Full validation (e.g. round-robin studies).

As full round-robin validation lies beyond the scope of CHIASMA, the project instead employs a structured collaborative validation approach during the pre-validation phase. This approach involves *pre-defined paired partners,* specific laboratory pairs working in close collaboration throughout the method transfer process. In each pair, one laboratory serves as the developer of the NAM, while the other functions as the testing partner. This setup allows controlled method transfer and emphasizes *real-time feedback* to refine documentation, identify bottlenecks, and improve experimental protocols.

1. Test Development and Optimization

CHIASMA partners focus on optimizing and refining *in vitro* NAMs to improve their capacity to predict long-term health effects. Key activities include:

• Addition and/or optimization of endpoints to enhance model performance
• Refinement of culture protocols to improve specificity and reduce the use of animal products
• Use of human-origin cells for increased relevance

To enhance the predictive capacity of NAMs for long-term outcomes, CHIASMA integrates molecular marker profiling into its framework. Since most *in vitro* systems are unsuitable for extended culture durations, the project seeks to link short-term exposures with long-term effects by identifying gene expression and epigenetic biomarkers.

Using a combined targeted and holistic approach, data-rich chemicals are evaluated based on known molecular targets, while data-poor substances undergo genome-wide analyses to identify novel markers. These biomarkers will be validated through high-throughput qRT-PCR, with technical support from expert partners. The same targeted strategy will be applied to CHIASMA’s key chemical classes—PFAS, (nano)pesticides, and 2D materials—to enhance model sensitivity and mechanistic relevance for long-term risk assessment.

To ensure transparency and harmonization, CHIASMA partners employ standardized documentation tools, including the ToxTemp template [83] and the SPSF, as described in OECD GD 211 and the Good In Vitro Method Practices (GIVIMP) guidance [84]. These tools promote systematic and transparent reporting on test system characterization, data integrity, and reproducibility.

2. Pre-Validation: Optimization, Transferability, Reproducibility, and Relevance

The pre-validation phase, based on OECD GD 34 [82] and supported by GD 211 [84], evaluates four core aspects:

• Optimization (intra-laboratory validation):
  Each partner refines its protocols and performs reproducibility testing within its own laboratory to establish robust experimental procedures and determine key parameters such as Benchmark Doses (BMDs) for selected substances.
• Transferability (Inter-laboratory validation):
  CHIASMA employs a structured partner model, pairing each NAM developer with a testing partner experienced in comparable systems. The transfer process includes the exchange of SOPs, ToxTemps, and supporting documentation, with clearly defined acceptance criteria for each critical step. This process evaluates not only technical feasibility and reproducibility but also documentation clarity, workflow practicality, and required expertise, allowing early identification of model-specific challenges and enabling tailored validation strategies.
• Reproducibility (inter-laboratory validation):
  When model readiness permits, both developer and testing partner perform parallel experiments using a shared set of reference chemicals. Comparative analyses ensure consistency of outcomes across laboratories and build confidence in method reliability.
• Relevance:

CHIASMA demonstrates the regulatory relevance of its NAMs by applying them to three representative chemical classes:

a) (Nano)pesticides
b) PFAS
c) 2D materials for energy applications

These categories encompass substances of high societal and regulatory concern, characterized by diverse chemical structures, significant technical value, and complex safety challenges. Selected compounds are data-rich, with well-documented profiles on toxicity, production, and environmental distribution, ensuring meaningful benchmarking. The data generated will contribute to the evaluation of CHIASMA NAMs within NGRA frameworks, demonstrating their applicability for regulatory decision-making.

3. Regulatory Alignment and Future Submission

CHIASMA’s validation activities are structured to facilitate future regulatory alignment and formal validation of the developed NAMs. Upon achieving sufficient readiness levels, selected NAMs will be prepared for submission through the SPSF process to the OECD WNT or to EURL ECVAM for (pre-)validation. These submissions will support the progressive acceptance of CHIASMA methodologies within international validation frameworks, contributing to the broader adoption of animal-free, mechanistically informed, and sustainable testing strategies in regulatory toxicology.

All CHIASMA partners will complete validation plans aligned with OECD GD 211 and GIVIMP [84], outlining each model’s status and next steps for regulatory submission. While full-scale round-robin trials are not included, CHIASMA will follow the OECD GD 34 [82] modular approach and draws from initiatives like Versaille Project on Advanced Materials and Standards (VAMAS) [85] to support retrospective validation. An Open Access strategy ensures transparency, independent evaluation, and broader regulatory visibility. Models will ultimately be prepared for submission to EURL-ECVAM to undergo independent scientific validation and to support their acceptance as regulatory-relevant NAM.

All data generated within CHIASMA are critically evaluated and aligned with regulatory requirements (e.g. presence of adequate supporting documentation), prior of their integration in the CHISMA framework and submission of results and methods to standardization bodies, such as OECD (WNT, Working Party on Hazard Assessment (WPHA), Advisory Group on Emerging Science in Chemical Assessment (ESCA)), International Organization for Standardization (ISO), European Committee for Standardization (CEN), and EURL-ECVAM. Whenever possible and relevant, the work from CHIASMA will be used to support the generation of TGs, GDs and regulatory dossiers.

Strategic roadmaps will guide standardization, incorporating EU Codes of Practice for valorisation and standardization, and supporting documentation quality (e.g. GLP-fication). Selected NAMs may be transferred to Contract Research Organizations (CROs) or spin-offs (e.g. DNTOX, INVITROLIZE) to enhance market readiness. CHIASMA will also generate R&I recommendations and engage regulators via workshops and summits, ensuring models are fit for regulatory uptake and long-term impact.

In the transferability studies, the coupled partners will document not only the technical feasibility but also identify bottlenecks and define acceptance criteria. This includes both “soft” factors, such as clarity of documentation, required personal-support, and ease of data interpretation, as well as “hard” factors, such as required technical skills, workflow reproducibility, and inter-laboratory variability. These insights will feed directly into the refinement of current models and will give direct output to the project consortium’s understanding of effective *in vitro* model validation.

The ultimate goal of CHIASMA is to prepare its novel models for regulatory validation and acceptance, specifically targeting submission to the EURL-ECVAM and the OECD. The OECD GD 34 framework [82] will be used for retrospective validation of the NAMs, ensuring that the methods meet regulatory standards for safety assessments. These submissions will undergo peer review and independent evaluations to confirm that they align with the required regulatory criteria.

Each CHIASMA partner will outline progress and define next steps toward regulatory submission. These plans, guided by GD 211 and GIVIMP principles [84], will ensure that the NAMs are developed in line with international expectations and meet the standards required for global regulatory acceptance.

### 3.2 In silico methods and models development

The CHIASMA project will employ a suite of computational (*in silico*) NAMs (Table 2) to advance chemical safety assessment through the integration of computational modelling and data-driven approaches with experimental *in vitro* NAMs. Together, these complementary methods aim to predict toxicity, bioactivity, and exposure outcomes, while reducing reliance on traditional animal testing.

**Table 2:** List of computational NAMs being developed in CHIASMA.

***Computational in silico NAMs***		
***Name of the NAM***	***Developer***	***Endpoints***
Point of Departures (PoDs) from OMICS	TAU	• Identification of molecular PoDs and MoA from transcriptomic data
AOP fingerprint	TAU HYU	• Derivation of mechanistic pathway signatures • AOP reconstruction from OMICS data • Prediction of MIE/AO
KG-based interference	TAU	• Semantic integration and automated reasoning from toxicological and mechanistic data
PBK models	NTUA	• Simulation of ADME processes • *In vitro* to *in vivo* extrapolation assessment and human-relevant exposure prediction • Reverse dosimetry • Risk assessment employing AOPs information
ML and Physics based models	NovaM	• Integrated data- and mechanism-driven modelling for toxicity prediction, property inference, and pathway analysis
AutoML and Physics based models	NovaM HYU	• Automated model generation and optimization for chemical and material safety applications
QSAR/ QSMAR models	NovaM NTUA	• Prediction of toxicological effects based on molecular structure and descriptors
Read-across/ Structural alters	NovaM	• Hazard inference based on chemical similarity and mechanistic analogs
LLM for Nanotoxicity Data Extraction	HYU	• Applications of Large Language Models to extract nanotoxicity data

The *in silico* NAMs combine cheminformatics, ML, and systems biology to analyse large and heterogeneous datasets, identify structure–activity relationships, and simulate complex biological interactions. When used in conjunction with *in vitro* models, these tools enhance the accuracy and efficiency of risk assessment, strengthen regulatory decision-making, and facilitate the design of safer and more sustainable chemicals and materials. Collectively, they provide efficient, reproducible, and ethically responsible alternatives to conventional testing methods, aligning with the principles of NGRA and the 3Rs.

### 3.3 Development of the CHIASMA knowledge graph and text mining tools

The integration of KGs and text-mining tools into the study of relationships between chemicals, materials, and biological effects, particularly when coupled with experimental *in vitro* NAMs, is emerging as a powerful approach for enhancing the predictive capacity of safety assessments. Text-mining tools enable the systematic extraction of information from scientific literature and databases, while KGs provide a structured framework to organize this information. Within CHIASMA, these technologies form the first digital layer of the CHIASMA SSbD Assessment, supporting data harmonization, interoperability, and reuse.

KGs are graph-based data structures in which nodes represent entities (e.g., chemicals, genes, cell lines, diseases) and edges denote the relationships between them. This interconnected architecture enables the integration, querying, and analysis of heterogeneous data from diverse sources, allowing the identification of complex relationships between chemical structures, biological pathways, and toxicological outcomes. As such, KGs offer a holistic approach to chemical safety assessment, providing a foundation for data interconnectivity, mechanistic reasoning, and reusability [86].

In the context of *in vitro* NAMs, KGs integrate diverse datasets derived from experimental systems, such as gene-expression changes, protein activity, and cellular responses, with chemical-specific characteristics including molecular structure, dose, and exposure route. This fusion enables broad and reliable predictions of how a given material or chemical compound may affect human health or the environment, leveraging both experimental evidence and accumulated knowledge from the literature.

Within CHIASMA, a dedicated KG is being systematically developed by integrating multi-source experimental and literature-derived data on gene expression, chemical–gene interactions, and *in vitro* models. This KG will serve as a foundational tool for defining the applicability domain of *in vitro* systems, identifying the range of chemicals and biological processes that each model can reliably evaluate.

The approach builds on CHIASMA’s ability to characterize steady-state gene-expression levels across different cell lines. By comparing baseline expression states and exposure-induced alterations, the relative responsiveness of individual genes to chemical perturbation can be quantified. This comparison allows identification of the most suitable cell lines for assessing specific combinations of chemical classes and biological processes linked to adverse outcomes. As the project evolves, the KG will expand through the integration of omics data generated from advanced co-culture NAMs. These complex systems, which differ markedly from monocultures in expression profiles, more accurately reflect tissue-level biological interactions and thus significantly enhance predictive power.

In the CHIASMA KG, nodes represent *in vitro* models (e.g., cell lines or advanced NAMs), genes, diseases, and chemicals. Edges connecting models and genes capture steady-state gene-expression values obtained from resources such as the Human Protein Atlas [87]. Edges linking chemicals and genes incorporate experimental data generated within CHIASMA, detailing transcriptional alterations induced by the project’s priority chemical classes, namely PFAS, 2D materials, and (nano)pesticides. Each association is weighted using results from differential and dose-dependent gene-expression analyses [88], [89], [90], which are increasingly recognized as powerful approaches to characterize exposure responses and mechanisms of action (MoA) leading to Adverse Outcomes (AOs) [91], [92]. Additional associations extracted from public databases such as the Comparative Toxicogenomics Database (CTD) [93] are also incorporated, with scores reflecting the relevance or strength of each chemical–gene link.

The resulting KG supports the development of a scoring system to prioritize chemical–test system pairs, based on the structural similarity and physicochemical properties of untested chemicals relative to those already characterized. It also enables evaluation of transcriptomic similarity between baseline *in vitro* systems and dysregulation patterns induced by new substances. Users can refine prioritization dynamically according to specific exposure scenarios. Over time, the KG will evolve into an expanding knowledge base enriched with new omics datasets, progressively extending the applicability domain of CHIASMA’s test systems.

In its current form, the CHIASMA KG integrates a large body of interconnected data structured into triplets (e.g., *cell line–gene–chemical* or *cell line–gene–disease* relationships) linked by gene-expression signatures. From differential expression data, general scores are derived for each cell line–chemical or cell line–disease pair, quantifying model responsiveness and predictive potential. These scores are directly connected to KEs within AOPs, enabling mechanistic interpretation of observed transcriptomic perturbations.

As new transcriptomic signatures from CHIASMA NAMs are integrated, the KG will enable the inference of NAM–chemical and NAM–disease associations, expanding biological applicability domains. Beyond quantifying association strength among biological entities, the graph structure allows exploration of emergent patterns, such as how specific chemical classes cluster with particular cell types or phenotypic outcomes, and how tissue-related models exhibit shared response profiles. Node labelling within the KG supports exploration across multiple layers of information (e.g., chemical MeSH classes, cell types, tissues), facilitating flexible grouping by relevant biological or chemical features.

**Fig. 5:**
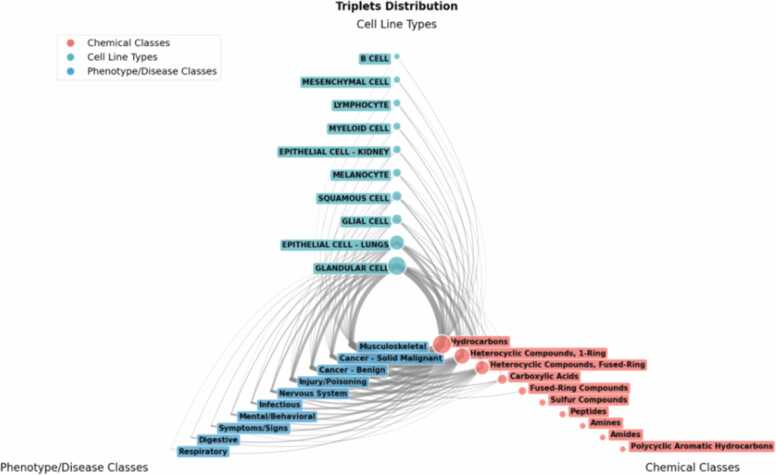
Graphical representation of the underlaying concept of the CHIASMA KG. Cell lines are grouped by type, chemicals are grouped by the second level of the Chemical MeSH hierarchy, and diseases are grouped by the ICD11 chapters. The node sizes represent the number of triplets (chemical – cell line - disease) that class is involved in, and edges between them reflect the frequency of triplet occurrences, with edge width indicating the volume of associations.

A preliminary visualization of the CHIASMA KG is shown in Fig. 5. This explorative tool enables prioritization of biological systems with strongly inferred and mechanistically plausible Modes of Action (MoAs) for further investigation, while preserving the *in vitro* context. Ultimately, the CHIASMA KG provides both objective, data-driven recommendations for selecting the most responsive cell models for specific chemical or disease assessments and mechanistic explainability of predicted relationships. By linking transcriptomic perturbations, key events, and genetic annotations, the KG forms a cornerstone of CHIASMA’s integrative framework for NGSA.

The integration of the KNIME (Konstanz Information Miner) workflow, illustrated in Box 1, using the Enalos+ KNIME nodes [94], [95], plays a pivotal role in enabling the systematic retrieval, structuring, and analysis of data for inclusion in the CHIASMA KG. KNIME is an open-source data analytics and workflow platform that facilitates automated data preprocessing, integration, and analysis through modular and reproducible pipelines.

Box 1 **Data Integration into CHIASMA KG workflow.**

The initial step of the workflow involves the Table Creator (Node 31) and Main PubChem node (Node 32), which retrieve detailed molecular descriptors, physicochemical properties, and standardized chemical identifiers (SMILES, InChIKeys). This data form the foundation for subsequent cheminformatics analysis, ensuring that the CHIASMA KG contains well-structured, machine-readable molecular information. A critical component of the workflow is chemical similarity analysis, which supports the identification of structurally related compounds with potentially comparable bioactivity and toxicity profiles. The Similarity node (Node 41) identifies chemically similar substances, while the Assay node (Node 43) retrieves bioassay data, capturing structure-activity relationships that can be mapped to toxicogenomic alterations within the KG. The Vendor node (Node 42) then determines the commercial availability of these chemicals, facilitating further in vitro validation.

The bioactivity profiling and toxicity assessment pathway of the workflow further enriches the CHIASMA KG by linking chemical exposures to molecular changes. The Assay node (Node 37) retrieves bioactivity data related to the input compound, which is subsequently categorized by the Assay Class node (Node 40) as active or inactive. The SID node (Node 38) converts Substance Identifiers (SIDs) to Compound Identifiers (CIDs), allowing for the seamless integration of experimental toxicology data with literature-based evidence. The Vendor node (Node 39) identifies commercially available bioactive compounds, ensuring that relevant chemicals can be studied in CHIASMA’s in vitro models. Additionally, patent mining and regulatory analysis are incorporated into the CHIASMA KG through this workflow. The Patent node (Node 33) retrieves patents associated with the input compound, while the Patent to SID node (Node 34) extracts SIDs from these records, allowing the identification of patented compounds relevant to CHIASMA’s research. The SID node (Node 35) converts these SIDs into CIDs, enabling further cheminformatics analysis, and the Main PubChem node (Node 36) retrieves detailed molecular information on patented compounds, contributing to the regulatory and innovation components of the CHIASMA KG.

**Figure:**
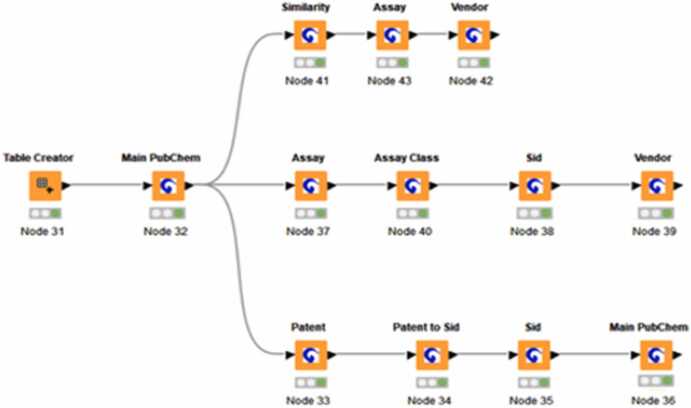
Workflow for integrating checmial, bioactivity, and regulatory data into the CIHASMA Knowledge Graph. The Table Creater (Node 31) and Main PubChem node 8Node 21) provide foundational molecular information. Three branches enrich the KG: (1) chemical similarity, bioassays, and vendor availability (Node 41-42); (2) bioactivity profiling assay classification, DIS-CID mapping, and vendor lookup (Nodes 37-39); and (3) patnet retrieval, SID extraction, and detialed compound information (Nodes 33-36).

Within the CHIASMA framework, this workflow allows the efficient extraction and organization of publicly available datasets, including chemical properties, bioactivity data, and Structure–Activity Relationships (SARs), from key repositories such as PubChem, ChEMBL, and PubMed. By harmonizing and curating these diverse data sources, KNIME ensures that relevant molecular and toxicological information is readily accessible and standardized for downstream predictive modelling and safety assessment within the CHIASMA KG.

The initial stage of the KNIME workflow involves the Table Creator (Node 31) and Main PubChem node (Node 32), which retrieve detailed molecular descriptors, physicochemical properties, and standardized chemical identifiers (e.g., SMILES, InChIKeys). These data form the foundation for subsequent cheminformatics analyses, ensuring that the CHIASMA KG is populated with well-structured, machine-readable molecular information.

A key component of the workflow is chemical similarity analysis, which enables the identification of structurally related compounds that may exhibit comparable bioactivity and toxicity profiles. The Similarity node (Node 41) identifies chemically similar substances, while the Assay node (Node 43) retrieves corresponding bioassay data, capturing structure–activity relationships (SARs) that can later be mapped to toxicogenomic alterations within the KG. The Vendor node (Node 42) determines the commercial availability of these substances, facilitating their inclusion in CHIASMA’s *in vitro* testing pipeline for experimental validation.

The bioactivity profiling and toxicity assessment component of the workflow further enriches the CHIASMA KG by linking chemical exposure data to molecular-level biological changes. The Assay node (Node 37) retrieves bioactivity data related to each compound, which is then classified by the Assay Class node (Node 40) as *active* or *inactive*. The Substance Identifier (SID) node (Node 38) converts SIDs into Compound Identifiers (CIDs), enabling the seamless integration of experimental toxicology data with literature-derived evidence. The Vendor node (Node 39) identifies commercially available bioactive compounds, ensuring that priority substances can be studied experimentally in CHIASMA’s *in vitro* models.

In addition to chemical and bioactivity information, patent mining and regulatory analysis are incorporated into the workflow to enhance the regulatory and innovation dimensions of the CHIASMA KG. The Patent node (Node 33) retrieves patent records associated with each compound, while the Patent-to-SID node (Node 34) extracts SIDs from these records, facilitating the identification of patented substances relevant to CHIASMA’s research focus. The SID node (Node 35) converts these identifiers into CIDs, and the Main PubChem node (Node 36) retrieves detailed molecular and structural data for the patented compounds. Collectively, this ensures that the CHIASMA KG integrates scientific, commercial, and regulatory intelligence to support comprehensive and forward-looking chemical and material safety assessment.

### 3.4 Chemocentric framework for chemical safety and sustainability assessment

QSAR models will be integrated into the CHIASMA chemocentric framework to support comprehensive chemical safety and sustainability assessments. QSAR models are mathematical and statistical tools that predict the biological and toxicological properties of chemicals or materials, enabling efficient, accurate, and animal-free evaluations. They provide quantitative predictions of critical toxicological endpoints based on computational descriptors and/or experimental parameters, thereby reducing reliance on conventional testing.

The development of robust QSAR models within CHIASMA involves the systematic curation of high-quality datasets, the training of predictive algorithms using advanced statistical and ML methods, and external validation to assess model accuracy, reliability, and domain of applicability. Each model will include a clearly defined Applicability Domain (AD) to delineate its operational boundaries and strengthen confidence in its predictions. All models will ultimately be deployed as user-friendly web services, featuring both a Graphical User Interface (GUI) for intuitive access and an Application Programming Interface (API) for seamless integration into computational workflows and regulatory pipelines.

To further enhance modelling efficiency, CHIASMA is developing an Automated Machine Learning (AutoML) framework, powered by the Isalos Analytics Platform. This system automates critical modelling steps such as feature selection, hyperparameter optimization, and algorithm selection, ensuring optimal predictive performance across diverse applications in nano-safety and nanomaterial risk assessment. The AutoML framework is also being expanded to integrate physics-based models, combining domain-specific knowledge with first-principles (ab initio) approaches to improve both accuracy and mechanistic interpretability.

A key application area of this methodology is the prediction of nano-fertilizer properties, where experimental data and mechanistic models are systematically combined to optimize formulations for maximum efficacy and minimal environmental impact. These activities are embedded within the broader CHIASMA framework, emphasizing chemocentric, multi-layer, and read-across modelling strategies.

Through the development of QSAR and Quantitative Structure–Mechanism-of-Action–Activity Relationship (QSMAR) models, CHIASMA employs advanced ML and AI approaches to link structural features to biological activity and MoA. This integrated approach establishes a comprehensive, mechanistically informed framework for SSbD innovation, accelerating hazard prediction and supporting NGRA objectives.

### 3.5 PBK modelling within CHIASMA

PBK models are *in silico* tools used to predict the ADME of substances in humans and environmental species. These models rely on mathematical representations of physiological, chemical, and biochemical processes to simulate concentration–time profiles within organs and tissues under various exposure scenarios [96]. By incorporating factors such as tissue permeability, protein binding, and enzyme-mediated metabolism, PBK models provide a dynamic, mechanistic understanding of ADME processes and the determinants of internal exposure [97].

Within the CHIASMA project, PBK models are being developed using a middle-out approach, integrating available *in vivo* data from the literature with *in vitro* measurements to fill existing data gaps. Specifically, *in vitro* assays will be employed for parameterization after appropriate in vitro–*in vivo* extrapolation (IVIVE) scaling. Cellular uptake kinetics will also be quantified and incorporated into the models by measuring concentration data in the recipient compartment medium at multiple time points, as well as intracellular concentrations in cell lysates [98]. The combined use of *in vitro* and *in silico* workflows has been demonstrated to effectively predict *in vivo* biokinetic behaviour [99].

The inclusion of human-specific physiological and anatomical parameters, such as glomerular filtration rate, regional blood flows, tissue volumes, and surface areas, further enhances model relevance and interpretability. This integration supports a mechanistic and physiologically grounded framework that improves confidence in model predictions. Sensitivity analyses are used to quantify the relative contribution of individual parameters to overall model outcomes, providing a systematic measure of parameter importance and uncertainty. The concordance between model predictions and human *in vivo* reference data, such as plasma concentration–time profiles, serves as a key criterion for evaluating the adequacy of *in vitro*-based parameterization.

The ultimate objective is to enable accurate estimation of safe human exposure levels while minimizing animal testing [100]. PBK model development within CHIASMA thus addresses a critical need by bridging the gap between *in vitro* and *in vivo* systems through biokinetic extrapolation, creating a robust computational framework for translating laboratory results into human-relevant exposure scenarios.

A central feature of this framework is the application of reverse dosimetry, which estimates the externally applied dose required to produce an internal tissue concentration equivalent to the *in vitro*-derived Point of Departure (PoD) [101]. Depending on the available data and substance properties, different toxicological dose metrics can be used to estimate *in vitro* PoDs, the most common being:

• Nominal concentration: the initial concentration of a test compound introduced into the experimental system, without accounting for bioavailability-altering processes such as degradation, adsorption, or binding. It reflects the theoretical exposure dose but not necessarily the biologically active concentration.
• Free concentration: the fraction of the compound unbound to medium constituents, representing the portion of the chemical that can freely interact with or enter cells, typically a more accurate measure of bioavailable dose.
• Intracellular concentration: the concentration measured within the cells themselves, directly reflecting the internal dose at the potential site of action.

**Fig. 6:**
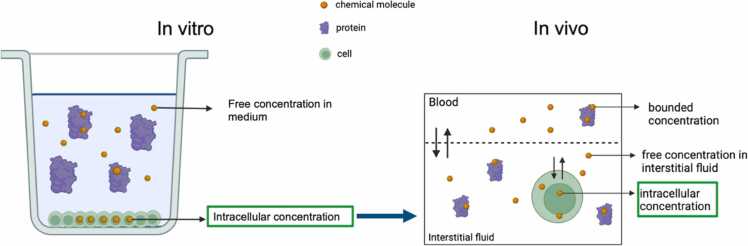
Schematic representation of different states a chemical can exist within an in vitro assay (right) and in a tissue in vivo, as simulated by a permeability-limited PBK model that includes blood, interstitial fluid, and intracellular compartments (left). The chemical molecules are present in different states: bound to proteins (bounded concentration); free in medium/interstitial fluid (free concentration); inside the target cells (intracellular concentration). The figure was created with BioRenderⓇ (biorender.com/).

These dose metrics can be determined experimentally or predicted computationally using *in silico* approaches [102]. For the *in vivo* analogues, PBK models simulate corresponding concentrations depending on model granularity. The nominal concentration is compared to simulated whole-tissue concentrations, the free medium concentration to the free tissue concentration, and the intracellular concentration to the predicted intracellular compartment concentration in the PBK model (Fig. 6).

**Fig. 7:**
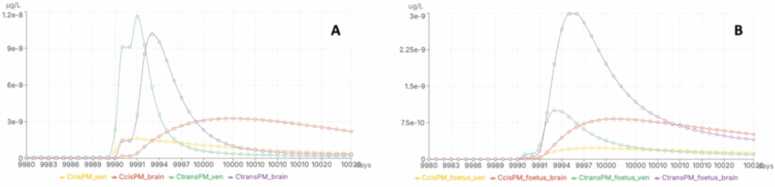
Maternal (A) and foetal (B) internal concentrations of cis- and trans-PM following an ingestion of 22.6 ng/kg bw/day of PM (PM median exposure from the Elfe cohort population). Y-axis is the concentration in µg/L and X-axis is the time in days. CcisPM_ven and CtransPM_ven: venous concentration of cis- and trans-PM in the pregnant women, CcisPM_brain and CtransPM_brain: brain concentration of cis- and trans-PM in the pregnant women, CcisPM_foetus_ven and CtransPM_foetus_ven: venous concentration of cis- and trans-PM in the foetus, CcisPM_foetus_brain and CtransPM_foetus_brain: brain concentration of cis- and trans-PM in the fetus.

By linking *in vitro* and *in silico* data through this harmonized PBK framework, CHIASMA enables the translation of mechanistic findings into quantitative risk assessment, advancing the development of NGRA and SSbD strategies.

Furthermore, PBK modelling enables the quantification of biological variability by incorporating physiological data derived from population-based distributions reported in the literature. This approach allows for a detailed assessment of interindividual differences in key kinetic parameters, such as tissue partitioning, metabolic rates, and renal clearance, across diverse population groups, including variations related to age, sex, and genetic background [103]. By capturing these dynamics, PBK models provide a comprehensive understanding of how variability in kinetic processes contributes to uncertainty in dose–response relationships and risk estimates. Importantly, this approach distinguishes between biological variability inherent to human populations and uncertainty stemming from other factors, such as *in vitro*-to-*in vivo* extrapolation or the derivation of *in vitro*-based Points of Departure (PoDs).

To demonstrate the application of PBK modelling within CHIASMA, a pregnancy-specific PBPK (p-PBPK) model developed for a mixture of pyrethroids-permethrin (PM), cypermethrin (CYP), and deltamethrin (DLT) [104], has been integrated into the CHIASMA SSbD Assessment platform. This model enables the characterisation of foetal exposure to pyrethroids resulting from maternal external exposure. Using a reverse dosimetry approach, maternal urinary metabolite concentrations from a population cohort [105] were used to estimate maternal exposure levels to the pyrethroids. The analysis indicated that none of the pregnant women in the cohort were exposed to PM, CYP, or DLT at levels exceeding existing toxicological reference values (TRVs) reported in the literature. However, when applying draft neurodevelopment-specific TRVs derived by Thépaut et al. (2024) [104], it was determined that approximately 2.5 % of the population exhibited exposure to PM exceeding the neurodevelopmental TRV, suggesting a potential risk for foetal neurodevelopment.

The model predicts both maternal and foetal internal concentrations, illustrating the differential distribution and accumulation of pyrethroids in foetal versus maternal tissues. Beyond quantifying foetal exposure, the model can also differentiate the contribution of individual pyrethroids to shared metabolites, as these compounds yield common metabolic products.

As an illustrative example, Fig. 6 presents predicted blood and brain concentrations of PM in a pregnant woman and her foetus following 24 hours of exposure to the median PM intake (22.6 ng/kg bw/day) estimated for the Elfe cohort, based on maternal urinary metabolite concentrations [104]. This exposure level lies well below the permethrin TRV of 20 µg/kg bw/day [106]. In the simulation, the virtual subject was a 27-year-old woman weighing 75 kg at the onset of pregnancy, with calculations performed for 20 weeks of gestation (equivalent to 9.989 days in model units). Predicted cis-PM concentrations were lower than trans-PM concentrations, consistent with the typical isomeric composition of commercial permethrin (approximately 40 % cis- and 60 % trans-isomer). Maternal concentrations were higher overall than those predicted for the foetus. In the foetal brain, the maximum trans-PM concentration was roughly three times higher than in foetal blood, while the maximal maternal trans-PM concentrations in blood and brain were approximately equal (1.16 × 10⁻⁸ µg/L in venous blood and 1.02 × 10⁻⁸ µg/L in brain).

This case study demonstrates how PBK models, particularly pregnancy-specific frameworks, can bridge exposure data, biomonitoring information, and toxicological thresholds, supporting mechanistic, population-relevant risk assessments that align with NGRA principles.

### 3.6 Integration of software- & data-handling & FAIRification

The integration of the diverse methods and tools described above into the CHIASMA SSbD Assessment places significant demands on data harmonization and interoperability. To meet these requirements, all data and models within CHIASMA undergo FAIRification, that is, they are made Findable, Accessible, Interoperable, and Reusable (FAIR). This process is essential not only for future reuse and knowledge sharing, but also for ensuring seamless primary application of the tools within the CHIASMA IATAs and NAMs.

**Fig. 8:**
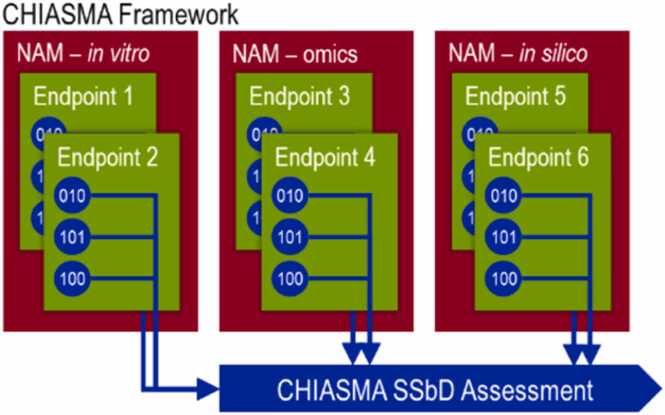
Relationship and combination of the different NAMs, IATAs, and endpoints into the CHIASMA SSbD.

FAIRification applies equally to experimental and computational data as well as to the models, workflows, and software used for their generation and analysis. To support this and ensure that all consortium partners operate under a unified framework of high-quality information management, CHIASMA is establishing a distributed knowledge infrastructure complemented by SSbD Assessment Stewarding Services (Fig. 8). This infrastructure ensures transparent data governance, enhances interoperability among project components, and supports the long-term accessibility and sustainability of CHIASMA’s digital ecosystem.

Knowledge provision for the CHIASMA SSbD Assessment platform, and for all CHIASMA activities more broadly, is built upon a distributed knowledge infrastructure combined with a central registration service. This system ensures that all research outputs are indexed for findability, accessibility, and interoperability, enabling their efficient use within CHIASMA and their eventual public reusability. Each dataset is accompanied by comprehensive provenance trails that document the origin and transformation of materials and samples across life cycle stages, as well as all data processing steps from raw data to final interpretation.

During the project’s design phase, it was recognized that a limited set of predefined data reporting templates would not provide sufficient flexibility to accommodate the wide range of data types generated by the diverse experimental and computational methods involved. Consequently, CHIASMA adopted a more adaptive and decentralized approach. All outputs are first integrated in their native formats, as produced according to the internal workflows of each partner organization and using domain-specific tools tailored to the nature of the data, for example:

• Electronic Lab Notebooks (ELNs) for experimental protocols and observations,
• data storage solutions implementing NAM-specific (meta)data schemas, and
• software repositories and version control systems for computational models and scripts.

Following initial integration, automated data transformation workflows are applied to produce harmonized datasets, aligning them with the information requirements defined at both institutional and project levels. These harmonization processes also ensure compliance with existing (meta)data standards and minimal regulatory reporting guidelines, facilitating interoperability with external databases and regulatory platforms.

**Fig. 9:**
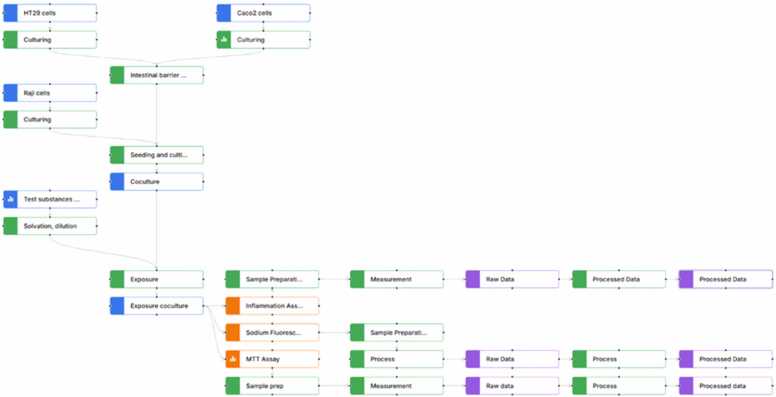
Part of the workflow for the preparation and exposure of the intestine barrier [61] in vitro model visualised in the form of an study design map [107]. Each of the boxes representing specific stages of the biological systems, materials and/or chemicals as well as transformations of these can be linked to a research output (protocols or data).

To ensure full transparency and traceability, CHIASMA employs Study Design Maps, an updated and extended version of Instance Maps [107], to visually represent experimental workflows, link generated data to the corresponding protocols, computational models, and software, and document material, sample, and data provenance. These visualizations (Fig. 9) provide a clear, navigable overview of the study design and data lineage, supporting reproducibility, regulatory credibility, and cross-disciplinary collaboration within the CHIASMA framework.

The harmonized data format, central registration service, and study design maps are integral components of CHIASMA’s strategy to achieve full FAIRness and computer-actionability of all its data, models, and software. Multiple FAIR Implementation Profiles (FIPs) are currently being developed or adapted from previous initiatives to guide partners in the selection of tools and workflows for implementing FAIR principles across different research objects. These FIPs ensure a consistent and harmonized approach among partners, and, equally importantly, across data, models, and software. This activity complements the project’s Data Management Plan (DMP) by translating its high-level guidance into clear, executable workflows.

To foster cross-project and cross-domain interoperability within the broader European digital ecosystem, CHIASMA actively collaborates and exchanges knowledge with other SSbD initiatives, including the Partnership for the Assessment of Risks from Chemicals (PARC) and, in the future, the Innovative Advanced Materials for Europe Partnership.

To ensure the adoption of robust data management and FAIRification practices across the consortium and by end-users of the CHIASMA IATAs/NAMs, the SSbD Assessment Stewarding Service coordinates all activities related to the collection, management, and governance of (meta)data. This includes gathering requirements from partners, defining (meta)data and application interface guidelines, and selecting or developing appropriate tools for data extraction, storage, and transformation. The data stewarding service also leads the decision process regarding security and privacy protection levels for each data type and supports the implementation of Open Science and FAIR principles for all public and private project outputs.

These efforts began with a stewardship workshop, which reviewed the existing data management practices of all partners, identified key requirements for harmonization and FAIRification, and introduced core concepts and tools for implementation within CHIASMA. The outcomes of this workshop now underpin individualized support activities, enabling each partner to align its data and workflows with the shared CHIASMA standards.

The CHIASMA SSbD Assessment CSI

The development of the CHIASMA SSbD Assessment CSI marks a major step toward streamlining and enhancing SSbD evaluations. Powered by the Enalos Cloud Platform and Jaqpot25, the CSI serves as a centralized digital entry point for both consortium members and external users to access CHIASMA’s SSbD Assessment framework and the associated IATAs and NAMs.

Through its Graphical User Interface (GUI), the CSI enables users to:

• retrieve existing datasets from the CHIASMA Knowledge Infrastructure or other compatible sources,
• upload new datasets compliant with FAIR data principles, and
• execute IATAs to explore, visualize, and interpret results.

By consolidating all relevant data and computational models, the platform provides users with a comprehensive view of the confidence levels associated with each assessment and identifies areas where additional data could reduce uncertainty. This integrated environment strengthens the foundation for data-driven regulatory evaluations by combining advanced computational methodologies with transparent, traceable, and reproducible workflows.

A significant milestone in this effort is the deployment of the CSI as an operational instance on the Enalos Cloud Platform, representing a major advancement in the realization of CHIASMA’s SSbD objectives. The cloud-based infrastructure enables efficient management of large and complex datasets, supporting both research and regulatory applications. By facilitating the execution of IATAs and delivering intuitive result visualizations, the platform promotes transparency, reproducibility, and interpretability in model predictions.

Researchers and regulators can now leverage these integrated computational workflows for the assessment of nanomaterials and chemical substances, directly supporting CHIASMA’s mission to enable data-driven safety and sustainability evaluations.

Aligned with CHIASMA’s FAIR data standards, the CSI allows users to upload, manage, and retrieve datasets from the knowledge infrastructure while maintaining data integrity and provenance. Its web-based architecture, enhanced with detailed documentation and API functionalities, ensures seamless incorporation of new computational models and interoperability with external databases and regulatory platforms. The inclusion of confidence metrics within the predictive models allows for quantification of uncertainty, guiding users toward areas where further data generation may refine conclusions.

By unifying data accessibility, model execution, and uncertainty quantification in a single environment, the CSI platform effectively bridges scientific research and regulatory application. It represents a cornerstone of CHIASMA’s commitment to transparent, reproducible, and interoperable digital infrastructures, supporting both the scientific community and regulatory bodies in the transition toward NGSA and SSbD paradigms.

Ongoing development of the CHIASMA CSI on the Enalos Cloud^2^ and the Jaqpot^3^ Platform will involve the integration of additional predictive models, building on examples such as the Iron The Carbide Nanoparticles (ICNP) cytotoxicity model (Box 2) will be further developed to expand its applicability across a broader spectrum of chemicals and nanomaterials, while undergoing continuous refinement to improve computational accuracy, robustness, and reliability. Dedicated efforts will focus on comprehensive model validation to strengthen regulatory confidence and refine methodologies for uncertainty quantification.

Box 2 **ML combination with Physics based models at the CHIASMA Instance of Enalos Cloud Platform.**

Among the newly deployed capabilities on the CSI platform is the atomistic descriptor-driven predictive model for assessing the cell viability impact of Iron Carbide Nanoparticles (ICNPs) https://enaloscloud.novamechanics.com/chiasma/icnp/. This model uses a random forest algorithm to forecast cytotoxicity following 24-hour exposure, incorporating detailed atomistic descriptors that capture critical aspects of nanoparticle behaviour. By providing reliable predictions of cytotoxicity outcomes, the tool supports regulators and researchers in evaluating the safety profiles of emerging nanomaterials in line with SSbD principles. Its integration into the Enalos Cloud Platform broadens the suite of available models and positions the CHIASMA instance as a comprehensive resource for nanomaterial risk assessment.

**Figure:**
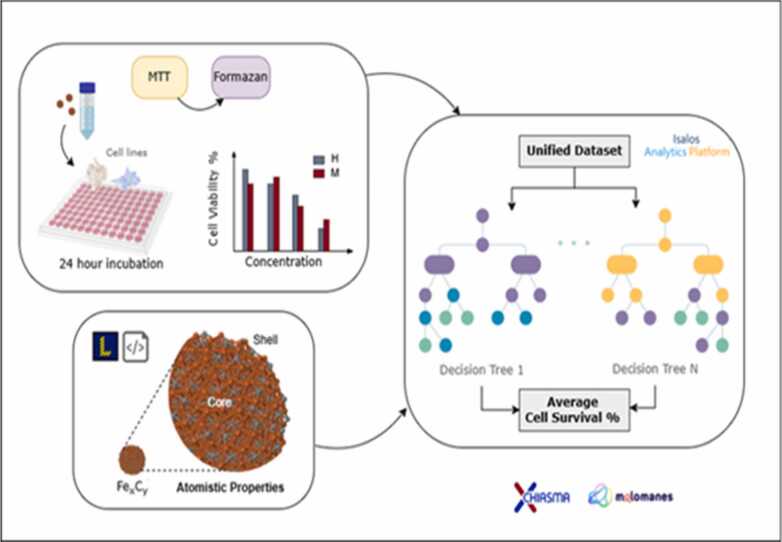
The CHIASMA instance of the Enalos Cloud Platform integrates an atomistic descriptor–driven model for predicting the 24-hour cytotoxicity of Iron Carbide Nanoparticles (ICNPs) using a random forest algorithm. The model captures key nanoparticle behaviors through detailed atomistic descriptors and provides reliable viability predictions aligned with SSbD principles. Its deployment expands the platform’s modelling capabilities and strengthens CHIASMA as a comprehensive resource for nanomaterial safety assessment.

In parallel, CHIASMA will prioritize the creation of user training resources, detailed documentation, and interactive guidance materials, ensuring that both researchers and regulators can efficiently access and apply the platform’s full range of functionalities. Through ongoing collaboration with policymakers, regulatory authorities, and standardization bodies, the CHIASMA SSbD Assessment CSI platform will continue to evolve in line with emerging standards, guidelines, and regulatory expectations.

By maintaining this iterative development and close regulatory dialogue, the CSI platform aims to foster broad adoption and implementation of SSbD principles, supporting the long-term goal of enabling transparent, evidence-based, and harmonized safety assessments across the European innovation ecosystem.

### 3.7 Methods integration & application

To advance chemical and material safety within the CHIASMA framework, it is essential to integrate the outputs of its various innovative methodologies into established assessment tools. Among these, LCA provides a structured and standardized foundation for evaluating the environmental impacts of products throughout their entire life cycle—from raw material extraction to end-of-life disposal. Widely used in chemical engineering and process development, LCA allows the comparison of the sustainability performance of conventional and emerging technologies through its four core steps:

1. Goal and Scope Definition,
2. Inventory Analysis,
3. LCIA, and
4. Interpretation [108], [109].

However, traditional LCA frameworks lack the capability to incorporate advanced toxicological assessments and mechanistic, risk-based safety evaluations [110], particularly for novel or poorly characterized substances. Recent research has begun to address this gap through the integration of ML and AI techniques [111]. In the context of SSbD, the AI-based LCA literature primarily explores two methodological pathways:

a) direct (end-to-end) impact prediction, where material or product representations are directly linked to predicted aggregate life cycle impacts across one or more categories [41], [112]; and
b) inventory prediction, where intermediate life cycle inventory (LCI) data are predicted and subsequently used in standard LCIA models [113], [114].

Building on this foundation, CHIASMA integrates principles of NGSA into LCA to bridge the gap between environmental impact assessment and mechanistic toxicological evaluation. NGSA represents a science-based, human-relevant, and predictive non-animal testing paradigm, combining *in vitro* and *in silico* methodologies for evaluating chemical and material safety across sectors such as chemicals, pharmaceuticals, cosmetics, and food.

A key element of this integration is the use of IATAs, which systematically combine data from multiple sources, including *in silico*, *in vitro*, and *in chemico* approaches, to support hazard identification, characterization, and risk assessment [115]. Within CHIASMA, IATAs serve as a structural framework for incorporating mechanistic toxicological data into environmental impact models, thereby aligning predictive toxicology with both scientific innovation and regulatory expectations.

Combining NGSA and IATA methodologies with LCA enables a more comprehensive, predictive assessment of both safety and sustainability. This integrated approach provides the ability to capture long-term human health effects alongside environmental performance, a critical capability when evaluating novel or data-poor substances. Nonetheless, several methodological challenges remain, chief among them the harmonization of data formats, the linkage of toxicological endpoints to environmental impact categories, and the adaptation of LCA databases that often lack CFs for advanced materials.

To address these challenges, CHIASMA supports the adaptation and expansion of existing LCA methodologies by translating outputs from *in vitro* and *in silico* workflows into new CFs, thereby extending the applicability of the USEtox model to novel substances. Traditionally built for molecular chemicals and largely based on animal-derived toxicity data, USEtox estimates human and ecosystem risks through fate, exposure, and effect modelling [116]. Transitioning toward *in vitro* and *in silico* inputs requires new extrapolation techniques, uncertainty analyses, and the development of substance-specific CFs, especially for advanced and nano-enabled materials [117].

Two complementary studies illustrate this transition. Salieri et al. [118] integrated SimpleBox4nano with USEtox, deriving nano-specific fate modelling within the USEtox framework. This approach provides implementable fate factors for engineered nanoparticles, enabling CF computation directly within standard LCIA workflows. Similarly, Romeo et al. [117] demonstrated a proof-of-concept for deriving *in vitro*-based human toxicity effect factors for inhaled, low-solubility nanomaterials. Their stepwise method incorporated *in vitro-to-in vivo* extrapolation to bridge dose metrics between cell-based assays and whole-organism exposures, thereby obtaining *in vitro*-derived human effect factors compatible with USEtox conventions and the broader LCIA framework.

The integration of these approaches enables the creation of an extended LCA methodology that can be reintroduced into the design and innovation phase, establishing an early feedback loop in which material parameters and functional modifications are iteratively screened for reduced human and environmental impact.

Despite remaining challenges, the integration of NGSA and IATA-derived data into LCA represents a transformative opportunity. It enables a truly holistic evaluation of chemical and material safety that aligns with SSbD principles and supports the early identification of safer and more sustainable alternatives. By embedding next-generation toxicological and mechanistic approaches into LCA, CHIASMA is helping to establish a predictive, robust, and sustainability-oriented framework for future chemical and material assessments, advancing both regulatory science and real-world protection of human health and the environment.

## 4 Discussion

Since the chemical and material innovation is constantly accelerating, there is a growing need for reliable, human-relevant methods to assess long-term safety predictions, particularly those concerning delayed and chronic toxicity, while minimizing reliance on animal testing. Traditional toxicological approaches are often inadequate for predicting chronic effects leaving critical gaps in current regulatory frameworks. To overcome these challenges, CHIASMA is advancing life-stage spanning human-relevant *in vitro* NAMs, that are aligning with 3 R principles. These models target essential biological systems, including external and internal barriers (skin, lung, intestine, placenta), metabolic organs (liver, kidney), and critical systems (brain, heart, reproductive and endocrine system). Each model is designed to generate data on endpoints relevant for regulatory risk assessment, including toxicity, sensitization, barrier integrity, and system-specific functions.

Importantly, regulatory relevance and acceptance of these models relies on their ability to address long-term safety predictions, particularly for chronic and delayed toxicity, which is a known gap in current regulatory frameworks. CHIASMA emphasizes the development of life stage-spanning NAMs and mechanically informed workflows aligned with AOP concepts. These efforts aim to identify and quantify critical elements such as MIEs, KEs, and KERs (Key Event Relationships) that drive long-term health outcomes. In parallel, a set of *in silico* tools is being developed to complement experimental data, supporting regulatory relevance and promoting the sustainable assessment of next generation chemicals and materials.

In the broader European landscape, PecisionTox, RISK-HUNT3R, and ONTOX stand as flagship Horizon Europe/H2020 projects that collectively form the foundation of the EU’s Next-Generation Risk Assessment (NGRA) vision. Each tackles a key layer of the scientific and regulatory transition toward animal-free, mechanism-based safety science.

PrecisionTox advances mechanistic discovery through *evolutionary toxicology*, mapping conserved toxicity pathways using multiple non-mammalian species complemented by human cell lines. It provides fundamental insight into the biological basis of toxicity but remains upstream of regulatory application due to uncertainties in cross-species extrapolation.

RISK-HUNT3R focuses on building the integrative framework for NGRA. Through its ASPA workflow, it unites toxicokinetics, in vitro hazard data, exposure modelling, and quantitative AOPs into a modular, weight-of-evidence decision system. It provides an operational blueprint for regulatory risk assessment, though many of its assays are still based on monocultures or single-organ systems**.**

ONTOX, in turn, targets the complex challenge of systemic repeated-dose toxicity by linking mechanistic, kinetic, and in vitro data via ontology-driven AI. Its focus on the liver, kidney, and brain advances chronic toxicity prediction, though its biological models remain largely 2D monocultures, limiting physiological realism.

Against this background, CHIASMA uniquely contributes by integrating advanced, human-relevant *in vitro* models directly into the SSbD and REACH/CLP regulatory frameworks. Unlike the other projects that rely heavily on either non-mammalian organisms (PrecisionTox) or simplified 2D cultures (RISK-HUNT3R, ONTOX), CHIASMA applies organ-level and multi-organ 3D human cell systems, such as organ-on-chip and co-culture models that replicate physiological barriers and life stage complexity. These systems capture chronic and delayed effects with higher fidelity, closing the translational gap between experimental evidence and regulatory decision-making.

furthermore, CHIASMA pairs its advanced biology with digital and regulatory innovation. FAIR-compliant “Instance Maps” ensure transparent traceability of experimental workflows, AI-assisted nanotoxicity data extraction accelerates evidence synthesis, and integrated testing strategies (ITS) are developed for regulatory case studies on PFAS, nano-pesticides, and 2D materials. In this way, CHIASMA acts as a regulatory bridge, converting the scientific advances of PrecisionTox, RISK-HUNT3R, and ONTOX into usable, interoperable, and dossier-ready frameworks**.**

CHIASMA’s unique strength lies in combining physiological complexity with regulatory pragmatism, transforming next-generation *in vitro* systems into actionable, reproducible, and FAIR evidence for sustainable chemical innovation and risk assessment.

### 4.1 External barrier models: skin, lung, intestine

CHIASMA is dedicated to advance the prediction of chronic exposure effects by developing human-based barrier models. For instance, one major focus is on respiratory sensitization, a major concern due to its association with occupational exposures and long-term respiratory diseases such as asthma [119] and currently lacking validated test methods. The ALIsens® model, a 3D alveolar lung *in vitro* model developed by LIST [59], provides a reliable alternative to animal studies thereby playing a crucial role in addressing the current gaps in hazard assessment. Traditional animal models often fail to recapitulate the anatomical and physiological characteristics of the human respiratory tract, challenging the accurate prediction of sensitization risks [120]. Additionally, the mechanisms of respiratory sensitization remain poorly understood, making it difficult to classify and regulate hazardous substances effectively [121], [122].

Although the EU’s CLP Regulation explicitly includes respiratory sensitization as hazard class requiring classification and labelling when sufficient evidence is available, there is currently no standardised and validated test method for this endpoint. CLP guidance therefore indicates that classification should be based on human evidence, epidemiological studies, and other relevant information. Because of this lack of validated methods, major regulatory frameworks such as REACH do not routinely request dedicated testing for respiratory sensitization during product registration or authorization. This creates a significant regulatory gap and is particularly concerning in occupational settings where workers are frequently exposed to respiratory sensitizers. Early identification and differentiation of respiratory sensitizers are crucial for implementing effective risk management and protecting human health. Regulatory agencies depend on robust data to enforce classification and labelling guidelines, ensuring the safe handling and use of sensitizing substances. The ALIsens® model is designed to generate mechanistic and predictive data that could support classification and labelling under current and future regulatory framework. Its validation and transfer across laboratories within CHIASMA will support its future acceptance by the OECD, thereby enabling its integration into risk assessment for occupational and consumer inhalation exposures as well as into regulatory frameworks. By providing mechanistic insights into early biomarkers of respiratory sensitization and long-term adverse effects, the ALIsens® model can enhance the understanding of sensitization mechanisms and improve hazard classification.

Building upon the ALIsens® model, a 3D alveolar lung *in vitro* model developed by LIST [63], HYU has adapted a new approach, single-cell mass cytometry (CyTOF) to investigate cell type- and cluster-specific heterogeneity in silver nanoparticle (AgNP) responses. This approach addresses a critical limitation of conventional toxicity assessments, which often rely on oversimplified in vitro systems and bulk population measurements that mask differences among individual cells. These findings provide unprecedented mechanistic insight into nanoparticle–barrier interactions, directly supporting CHIASMA’s goal of capturing biological complexity in hazard identification and contributing to the foundation for integrating single-cell data into future regulatory and safety assessment frameworks.

While the respiratory system is a primary entry route for airborne substances, the gastrointestinal tract plays a crucial role in the absorption and metabolism of various agents as well, that could cause health issues like inflammatory bowel disease [123], [124]. To address this scientific need, a human-based intestinal co-culture model has been developed at Empa to assess material and chemical impacts on intestinal barrier integrity, viability, and inflammation [61]. This model represents a comprehensive, human relevant approach to assessing the safety of ingested compounds and is being further verified to facilitate its potential recognition by the OECD. Its incorporation into regulatory frameworks can offer crucial data on how chemicals and materials affect amongst other endpoints intestinal barrier integrity and inflammation.

Similarly, for dermal exposures, CHIASMA builds on the already accepted OECD TG 442D for skin sensitization and toxicity to assess hazards effects of dermal contact that could result in skin diseases such as allergic contact dermatitis [125] therefore being relevant for occupational health and consumer safety.

### 4.2 Life-stage spanning and reproductive health models

Addressing developmental and reproductive toxicity, CHIASMA aims to fill a critical gap in long-term safety assessment for the next generation by developing placental and embryonic co-culture models. Prenatal exposure to chemicals is of particular concern due to the high vulnerability of the developing fetus and the potential for priming of (chronic) diseases in later life. Current developmental toxicity testing relies on studies conducted in pregnant animals; however, these studies are limited by significant species differences and the inability of animal models to accurately replicate human pregnancy disorders. Therefore, a human placenta model that recapitulates the key barrier layers will be developed and validated by CHIASMA partners for the assessment of the impact of chemicals and materials on placental translocation and function. This model is crucial for understanding the effects of advanced materials at the maternal-fetal interface, given the placenta's vital role in fetal development [126]. To further estimate early embryotoxicity and identify early biomarkers for long-term adverse pregnancy outcomes, a co-culture model of the placenta barrier and a human iPSC-derived embryoid body will be developed and used to examine the potential developmental effects of various chemicals. These models address limitations of animal-based developmental toxicity tests and are particularly relevant for understanding the impact of chemicals on reproductive health, with direct implications for implementing Directive (EU) 2022/431 on the protection of workers reproductive health [127].

For neurotoxicity, The BrainSphere (BS) model developed at the IUF [76] offers a life stage-spanning 3D *in vitro* platform simulating several life stages from proliferating neural progenitor cells (embryonic phase) to matured and synchronized neuron-glia networks (adult phase) over 7 weeks *in vitro*, thus enabling the assessment of neuronal developmental and aging. In CHIASMA, BS is used to assess long-term effects of chemical exposure, particularly in relation to the prevalence of Parkinson’s disease (PD). The model’s specific exposure strategies allow for the analysis of test compound concentration- and exposure time-dependent alterations in neuronal transmission using Micro Electrode Arrays (MEAs) and on the activation of e.g. astrocytes and microglia using Immunocytochemistry (ICC). The applicability of the 3D BS model has been demonstrated through its integration with spike-sorting techniques to assess individual neuronal subtypes transmission. For instance, the human multi-neurotransmitter receptor (hMNR) assay [76] has successfully employed the BS model to evaluate acute neurotoxicity using subtype-specific model compounds for glutamatergic, Gamma–Aminobutyric Acid (GABA)ergic, dopaminergic, serotonergic, and cholinergic transmission. Additionally, case study chemicals such as emamectin and trimethyltin chloride have further validated its utility. In CHIASMA, the scope of the hMNR assay will be extended to assess chronic effects of compounds on the emergence of PD-like long-term effects, particularly impaired dopaminergic transmission and altered microglia activation [128]. By replicating key aspects of human brain functionality and PD progression in a life-stage spanning model, BS outperform traditional, short-term exposure *in vitro* models in detecting chronic and delayed neurotoxic effects. Moreover, ongoing research and discussions on studying PD pathogenesis using human Midbrain-like Organoids (hMLOs) [129] further expand the scope of such NAMs. Long-term risk assessment and the elucidation of MoAs are essential components for supporting NGRA frameworks.

In addition to neuronal models, the BBB plays a central role in determining neurotoxic outcomes. The OECD has identified the BBB as a major gap in the current DNT *in vitro* test battery. The BBB is critical for compound kinetics, as it regulates how much of a substance actually reaches the brain. At the same time, chemicals can alter BBB functionality itself, which in turn may influence neuronal activity through disrupted communication between endothelial and neural cells, altered cytokine release, or compromised barrier integrity. To address these aspects, CHIASMA integrates BBB *in vitro* models. In the Transwell system, chemicals applied to the blood-facing side are evaluated not only for their effects on brain endothelial cell viability and BBB integrity, but also for secondary effects on neurons cultured on the basolateral (brain) side. This dual readout enables the identification of both barrier disruption and downstream impacts on neuronal functionality, thereby providing complementary information for a more comprehensive neurotoxicity assessment.

### 4.3 Models for endocrine disruption and reproduction

The endocrine system, vulnerable to disruption at many levels, is another focal point within CHIASMA. This system is a complex network of glands, hormones, and receptors that mediates communication between the nervous system and a set of peripheral organs associated with essential physiological functions such as reproduction, immunity, metabolism, and behavior. EDCs may interfere with the endocrine system at multiple levels, including hormone synthesis, secretion, transport, metabolism, and elimination [130], making it extremely challenging to fully assess the consequences of human exposure. A central endocrine organ – the pituitary gland – integrates signals from the brain and regulates peripheral targets, including other glands, by secreting multiple peptide hormones into the bloodstream. While there are discrepancies in the fertility regulation via hypothalamic-pituitary-gonadal axis within vertebrates, the production and release of pituitary gonadotropins, Follicle Stimulating Hormone (FSH), and Luteinizing Hormone (LH), are evolutionary conserved across mammals and bony fish [131]. A novel *ex vivo* pituitary model from transgenic medaka fish cell lines (Oryzias latipes) allows a real-time assessment of EDC effects on hormone secretion pathways, particularly gonadotropin production (FSH, LH) and release.

In addition to the disruption of neuroendocrine regulation, EDCs may directly affect gamete quality and functionality, thereby reducing reproductive success by interfering with gametogenesis, gamete maturation, and fertilization competence. To capture these direct effects, an *in vitro* gamete model using fish sperm and oocytes from medaka is being developed to assess gametotoxicity, including viability, sperm motility and morphology, as well as oocyte and sperm functionality. Together, both the neuroendocrine and gamete models provide mechanistic resolution for the impact of relevant EDCs on both central endocrine regulation and peripheral reproductive endpoints.

### 4.4 Complementing NAMs with computational models

CHIASMA’s *in silico* models serve to integrate, refine, and translate findings from experimental NAMs using advanced computational approaches, including QSAR/ QSMAR, PBK, ML, and read-across methods. These tools allow for predictive toxicology and dose extrapolation while reducing the need for *in vivo* data. A key feature is the use of AutoML to support reproducibility, performance optimization, and model accessibility through platforms like Enalos and Jaqpot.

### 4.5 PBK models and exposure-driven predictions

PBK models developed within CHIASMA offer mechanistic translation from *in vitro* systems to human exposure contexts. They incorporate middle-out parameterization from *in vitro* data and literature, supporting reverse dosimetry and *In Vitro* to *In Vivo* Extrapolation (IVIVE)**.** These models consider intracellular, nominal, and unbound concentrations, improving the interpretation of *in vitro* results in terms of real-world human exposure. Moreover, population variability is captured to assess sensitive subpopulations, thereby distinguishing between uncertainty and true inter-individual variability in toxicokinetic responses.

### 4.6 KGs and mechanistic data integration

To contextualize biological findings, CHIASMA employs KGs built from experimental results, public databases, and literature screening. These KGs facilitate mode-of-action hypothesis, inform AOP construction, and aid in defining the applicability domain of NAMs. They are essential for chemical prioritization and mechanistic interpretation, providing a framework that supports regulatory transparency and scientific rigor.

### 4.7 NGSA-LCA integration: towards sustainability-bases risk assessment

An important innovation in CHIASMA is the integration of NGSA tools with LCA, creating a holistic safety and sustainability framework. This framework leverages *in vitro* and *in silico* methodologies from NGSA to provide mechanistic, exposure-driven insights through NAMs and *in silico* modelling, thereby complementing the LCA structured approach to evaluate environmental impacts across the chemical lifecycle. This integrated approach addresses existing limitations in animal-based risk assessment which rely on *in vivo* data and focus on hazard identification without predictive capabilities on novel chemicals [115]. Traditional LCA methods, while evaluating environmental impacts systematically, lack the capacity for advanced toxicological assessments [109]. However, methodological challenges remain. These include divergent data formats, missing CFs for emerging materials, and the need for standardized data integration approaches. CHIASMA addresses this through a FAIR-compliant data infrastructure, centralized via the CSI platform, which harmonizes data registration, metadata, and model interoperability for streamlined decision support.

## 5 Conclusion

CHIASMA represents a significant step forward in transforming chemical and material safety assessment by developing advanced human-relevant, life stage-spanning NAMs and integrating them with AOP-based mechanistic frameworks. By focussing on long-term and life stage-specific effects, CHIASMA addresses a major gap in traditional toxicology testing, especially the prediction of chronic and delayed toxicity, thereby offering scientifically robust alternatives that reduce dependence on animal studies. These tools are designed not only to meet evolving regulatory expectations, but also to provide practical solutions that can be readily implemented in industry and risk assessment practice.

The NAMs developed within CHIASMA are targeting key biological barriers, metabolic organs, and critical systems and are supposed to deliver mechanistic, endpoint-specific data relevant for regulatory decision-making. Their integration with *in silico* models can enhance the predictive capacity and will allow for more rapid, cost-effective evaluations of next-generation chemicals and materials. These innovations enable safer product design, streamlined testing strategies, and more agile regulatory processes.

Beyond methodological innovation, CHIASMA also highlights the importance of collaboration across sectors. Key takeaways from the project include the value of engaging diverse stakeholders ranging from industry and regulatory agencies to academic researchers to align objectives, share expertise, and co-create tools that meet real-world needs. This interdisciplinary approach is essential for building trust, accelerating regulatory acceptance, and maximizing the usage of NAMs across sectors.

Moreover, integrating sustainability principles and applying LCA frameworks throughout the development of safer chemicals ensures that safety considerations are embedded from the earliest design stages. This holistic approach reinforces that scientific innovation must go hand in hand with environmental responsibility and public health protection.

In summary, CHIASMA underscores that advancing chemical safety requires not only cutting-edge science, but also meaningful stakeholder engagement and sustainable thinking. These combined efforts are key to driving progress toward safer, more effective, and future-ready risk assessment strategies.

## CRediT authorship contribution statement

**Roland Hischier:** Writing – review & editing, Writing – original draft, Conceptualization. **Ishita Virmani:** Writing – review & editing, Writing – original draft. **Haralambos Sarimveis:** Writing – review & editing, Writing – original draft, Conceptualization. **Elisa Thépaut:** Writing – original draft, Writing – review & editing. **Marc Majó:** Writing – review & editing, Writing – original draft. **Lorenzo Capini:** Conceptualization, Writing – review & editing. **Paraskevi Papakyriakopoulou:** Writing – review & editing, Writing – original draft. **Periklis Tsiros:** Writing – review & editing, Writing – original draft. **Antreas Afantitis:** Writing – review & editing, Writing – original draft, Conceptualization. **Vasileios Minadakis:** Writing – review & editing, Writing – original draft. **Pamina Weber:** Writing – review & editing, Writing – original draft. **Simona Kavaliauskiene:** Writing – review & editing, Writing – original draft. **Amin Sayyari:** Writing – review & editing. **Angela Serra:** Writing – review & editing, Writing – original draft. **Martin Paparella:** Writing – review & editing, Writing – original draft, Conceptualization. **Winfried Neuhaus:** Writing – review & editing, Writing – original draft, Conceptualization. **Émilie Brun:** Writing – review & editing, Writing – original draft. **Tina Buerki-Thurnherr:** Writing – review & editing, Writing – original draft, Conceptualization. **Iseult Lynch:** Writing – review & editing, Writing – original draft, Conceptualization. **Brugger Beatrice Anna:** Writing – review & editing, Writing – original draft. **Lee Eunsoo:** Writing – review & editing. **Thomas E. Exner:** Writing – review & editing, Writing – original draft, Conceptualization. **Riju Roy Chowdhury:** Writing – review & editing, Writing – original draft. **Sorhun Duygu Turan:** Writing – review & editing, Writing – original draft. **Maja Brajnik:** Writing – review & editing. **Katharina Koch:** Writing – review & editing, Writing – original draft, Conceptualization. **Tae Hyun Yoon:** Writing – review & editing, Writing – original draft. **Adriana Scattareggia Marchese:** Writing – review & editing, Writing – original draft. **Steffi Friedrichs:** Writing – review & editing, Writing – original draft, Conceptualization. **Zayakhuu Gerelkhuu:** Writing – review & editing, Writing – original draft. **Luisa Campagnolo:** Writing – review & editing, Writing – original draft, Conceptualization. **Christian Seitz:** Writing – review & editing. **Romain Fontaine:** Writing – review & editing. **Dario Greco:** Writing – review & editing, Writing – original draft, Conceptualization. **Emma Arnesdotter:** Writing – review & editing. **Mette Helen Bjørge Müller:** Writing – review & editing, Writing – original draft, Conceptualization. **Valentina Lacconi:** Writing – review & editing, Writing – original draft, Conceptualization. **Nour Attar:** Writing – review & editing. **Zouraris Dimitrios:** Writing – review & editing, Writing – original draft. **Peter Wick:** Writing – review & editing, Writing – original draft, Conceptualization. **Jack Morikka:** Writing – review & editing, Writing – original draft. **Dimitris Mintis:** Writing – review & editing, Writing – original draft. **Andreas Tsoumanis:** Writing – review & editing, Writing – original draft. **Tommaso Serchi:** Writing – review & editing, Supervision, Project administration, Funding acquisition, Conceptualization.

## Declaration of Generative AI and AI-assisted technologies in the writing process

During the preparation of this work the author(s) used ChatGPT-4.0 in order to edit the language to improve readability, clarity, and precision. After using this tool/service, the author(s) reviewed and edited the content as needed and take(s) full responsibility for the content of the published article.

## Declaration of Competing Interest

The authors declare that the research was conducted in the absence of any commercial or financial relationship that could be considered a potential conflict of interest. The funders had no role in the design of the study, in the collection, analyses, or interpretation of data; in the writing of the manuscript; or in the decision to publish the results.

## Footnotes

1 https://www.epa.gov/assessing-and-managing-chemicals-under-tsca/national-pfas-testing-strategy?utm_source=chatgpt.com

2 https://www.enaloscloud.novamechanics.com

3 http://www.jaqpot.org/
